# Potential Pharmacological Properties of Triterpene Derivatives of Ursolic Acid

**DOI:** 10.3390/molecules29163884

**Published:** 2024-08-16

**Authors:** Vuyolwethu Khwaza, Blessing A. Aderibigbe

**Affiliations:** Department of Chemistry, University of Fort Hare, Alice Campus, Alice 5700, Eastern Cape, South Africa

**Keywords:** ursolic acid, natural product, triterpenoids, pharmacological activities, derivatives

## Abstract

Ursolic acid (UA) and its derivatives have garnered significant attention due to their extensive pharmacological activity. UA is a pentacyclic triterpenoid found in a variety of plants, such as apples, rosemary, thyme, etc., and it possesses a range of pharmacological properties. Researchers have synthesized various derivatives of UA through structural modifications to enhance its potential pharmacological properties. Various in vitro and in vivo studies have indicated that UA and its derivatives possess diverse biological activities, such as anticancer, antifungal, antidiabetic, antioxidant, antibacterial, anti-inflammatory and antiviral properties. This review article provides a review of the biological activities of UA and its derivatives to show their valuable therapeutic properties useful in the treatment of different diseases, mainly focusing on the relevant structure-activity relationships (SARs), the underlying molecular targets/pathways, and modes of action.

## 1. Introduction

Natural products are chemical compounds extracted or isolated from living organisms. The molecular structural diversity of natural products and their unique pharmacological activities have attracted the attention of medicinal chemists. Natural products and their bioactive molecules act as valuable resources for drug discovery in medicinal chemistry [1]. In recent decades, drugs derived from or inspired by natural products have significantly contributed to disease treatment [2]. Between 1981 and 2019, 1881 drugs were approved, with 71 (3.8%) being natural products and 356 (18.9%) resulting from semisynthetic modification of natural products [3]. The discovery of artemisinin stands out as a significant achievement in the development of natural products [4].

**Figure 1:**
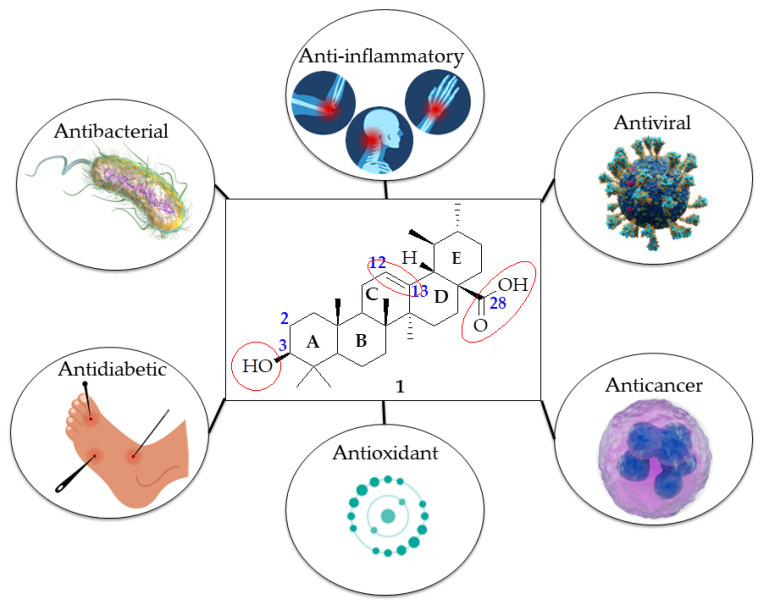
The chemical structure of UA with highlighted major active sites and its biological properties.

Triterpenoids, the largest group of natural bioactive molecules, have been widely explored due to their pharmacological properties [5,6,7]. The IUPAC name of UA (**1**) is 3-(β-hydroxy-urs-12-en-28-oic acid) and its chemical structure is shown in Figure 1. It is a pentacyclic triterpenoid of the ursane type, derived from various plant species. Historically, UA was first identified in the extract of apple epicuticular waxes during the 1920s [8,9]. Since then, numerous reports have detailed the isolation of UA from different plant species and the evaluation of its biological activities. Additionally, several studies have introduced new methods for developing novel formulations of UA and modifying its chemical structure to enhance its therapeutic effects in both in vivo and in vitro studies by improving its poor water solubility and bioavailability. In terms of biological activities, UA, with a basic chemical structure containing five 6-membered rings (A, B, C, D and E), has a remarkable variety of biological properties such as anticancer [10,11], antiviral [12,13,14], anti-inflammatory [15,16,17,18], anti-oxidant [19,20], antifungal [21,22,23,24,25], antibacterial [26,27], antidiabetic effects [28,29,30,31,32], etc. (as depicted in Figure 1).

In this review, we present an update on the structural modifications and therapeutic effects of UA and its derivatives on various infectious and non-infectious diseases. Additionally, we summarize the proposed mechanisms of action and molecular targets of UA and its derivatives.

## 2. UA Derivatization

The chemical structure of UA features three primary active sites for structural modification as highlighted in Figure 1. These sites include the carboxylic group at C-28, the β-hydroxy group at C-3, and an alkene between C-12 and C-13. These sites have been extensively explored for their potential pharmacological activities, with anticancer derivatives being among the most studied [33]. Modification of the C-28 carboxylic acid or C-3 hydroxyl group not only significantly enhances its biological activity but also decreases its toxicity [34,35]. In addition to the important role played by the C-28 amide and C-3 ester groups in inhibiting NF-κB, Jiang et al. reported the antitumor efficacy of long-chain diamine derivatives of UA through potential NF-κB inhibition. Derivatives with longer chain diamine side chains (n = 6) had better activity than those with shorter chains (n = 4 and 5). The presence of an O-acetyl substitution at C-28 proved to give more activity than the presence of a hydroxyl group at the same position [36]. Modifications have been employed in trying to change the oxidation state and /or the lipophilicity at C-3 [33]. Some studies have revealed that the configuration at C-3 plays a vital role in the antiproliferative activity of UA, and, at the same time, a free hydroxyl group at the same position decreases its anticancer efficacy [37]. Modifications such as the retention of the carbonyl at C-28 and the incorporation of several substituted aromatic rings at C-3 improved the anticancer activity of UA [38]. The introduction of a tetrazole moiety at the C-28 position of UA increases HIF-1α inhibitory activity while a bulky group at the C-3 position decreases the activity [39]. Wang et al. reported that compounds containing ester groups showed stronger antitumor activity towards MCF-7, HeLa and HepG2 cells than compounds with acylhydrazine, amide and carbonyl moieties. Though the introduction of N,N-dialkylamide decreased cytotoxicity activity, the compounds containing dimethylamino groups on the amide side chain displayed the strongest antitumor activity of all the derivatives indicating the role played by the groups on the amino side chain in enhancing cytotoxicity [40].

In summary, UA derivatization typically involves modifying specific functional groups on the UA structure. Common UA modifications include:

Esterification: introducing ester groups can improve lipophilicity and membrane permeability [41,42].
Amidation: converting carboxyl groups to amides can enhance stability and bioactivity [43,44,45].
Glycosylation: adding sugar moieties can improve solubility and bioavailability [46].
Oxidation/Reduction: modifying hydroxyl or carbonyl groups to influence activity and selectivity [47].
Acylation: adding acyl groups to enhance lipophilicity and cellular uptake [37].

## 3. Pharmacokinetic Studies

Although UA is recognized for its ability to inhibit the proliferation of various cancer cell lines, its pharmacokinetics are constrained by its low aqueous solubility. The effectiveness of cancer treatment relies on the drug’s ability to reach the tumor at therapeutic concentrations [48]. Preclinical trials have shown that UA is poorly absorbed through the intestines and rapidly eliminated by liver metabolism when orally administered. Intravenous administration of UA resulted in its diffusion throughout the body with non-specific distribution [49]. Since the oral route is considered better than the intravenous, efforts have been made to enhance the bioavailability of phytochemical antitumor agents following oral administration. The slow release of UA from UA-loaded nanoparticles resulted in lower cytotoxicity than the free UA [48]. Frolova et al. employed fluorescently labelled UA to track the penetration and distribution dynamics of UA in vitro. The confocal images after 12 h of incubation inferred the location of UA on the inner membranes (endosomes, Golgi apparatus and endoplasmic reticulum). After 18h, the labelled UA was bound to the mitochondrial receptors while the signal could be identified within the nucleus after 24 h [35].

Khan et al. determined that UA nano lipid vesicles (UALVs) and UA-loaded lipid vesicle gel (UALVG) exhibited distinct pharmacokinetic profiles following intranasal administration. UALVs showed a peak plasma concentration (C_max_) at 30 min (142.9 ± 5.49 ng/mL) with a T_max_ of 30 min, whereas in the brain, the T_max_ was 2 h (C_max_ 325.2 ± 20.86 ng/g). On the other hand, UALVG had a T_max_ of 2 h (C_max_ 184.73 ng/g) in plasma and 6 h (C_max_ 398.9 ng/g) in brain tissue. Penetration enhancement effects of the lipids and vesicular size meant that the T_max_ and C_max_ values would differ between the UALV and UALVG. The nanoformulation was non-toxic both to the nasal mucosa and the brain [50].

## 4. Biological Activities

UA is known for its diverse biological effects, such as its ability to combat cancer, diabetes, viruses, etc. (see Figure 1). Reviewing various reported studies, the sections below briefly introduce the pharmacological activities of UA and its derivatives.

### 4.1. Anti-Inflammatory Activity

Inflammation is the body’s natural response to a variety of stimuli, including pathogens, chemicals, and autoimmune triggers. It is essential for tissue repair and defense against these stimuli and is marked by symptoms like redness, pain and swelling [51]. Inflammation is an intricate process linked to the development of several diseases, such as cardiovascular conditions, cancer, neurodegenerative disorders, etc. [52].

UA’s mechanism of action involves an increase in proteins crucial for the terminal differentiation of keratinocytes, such as filaggrin, loricrin, and involucrin [53]. UA boosts intercellular lipids, especially ceramide, aiding in the restoration of the epidermal barrier [53,54]. Additionally, UA can reduce intracellular reactive oxygen species (ROS) production and mitigate the oxidative effects of UVB radiation by preventing lipid peroxidation [55,56]. The anti-inflammatory properties of UA are attributed to the suppression of NF- and the genes it regulates, including pro-inflammatory cytokines, Cyclooxygenase-2 (COX-2), and lipoxygenase [57].

Pentacyclic triterpenes are a highly potent class of natural products due to their diverse biological properties and structural variety [43]. UA, a plant-derived medicinal compound, targets different extracellular and intracellular mechanisms related to inflammation, angiogenesis, metastasis, and apoptosis. Moreover, UA synthetic derivatives have demonstrated good potential in disease prevention [52]. The anti-inflammatory activities of UA and its derivatives are attributed to their ability to inhibit histamine release from mast cells and suppress the activities of cyclooxygenase and lipoxygenase enzymes [53]. Additionally, they inhibit inducible nitric oxide synthase (iNOS) and elastase, reduce the inflammatory cytokine-induced expression of E-selectin on endothelial cells by preventing NF-kappa B (NF-κB) activation, and decrease the production of intracellular reactive oxygen species [58]. However, despite these benefits, UA faces some technological challenges, including low water solubility (~5.6 μg/mL), poor absorption, and low bioavailability, which limit its clinical potential [59].

**Figure 2:**
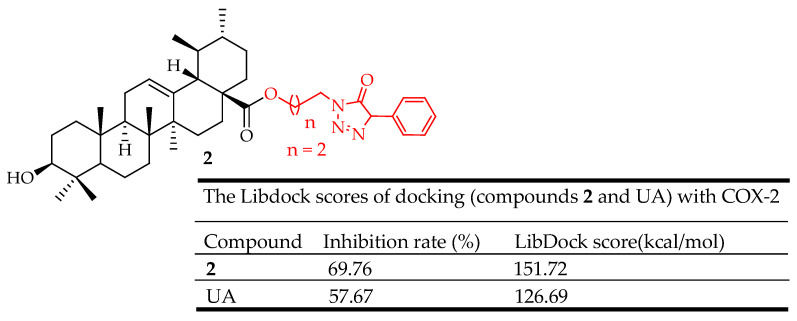
UA derivative 2 and its anti-inflammatory outcomes compared to UA.

Wei et al. [43] developed UA derivatives incorporating piperazine, triazolone, and oxadiazole groups to develop effective anti-inflammatory agents. Many of these molecules demonstrated significant anti-inflammatory effects at a dosage of 100 mg/kg. Notably, compound **2** (Figure 2) demonstrated a potent inhibitory effect on ear inflammation among all the synthesized molecules, with an inhibition of 69.76%, surpassing that of ibuprofen (25.17%) and indomethacin (26.83%) at a dosage of 100 mg/kg (i.p.), making it 2- and 3-fold more potent than these standard drugs used as control. The cytotoxicity of the derivatives was evaluated using the MTT assay, and none exhibited significant cytotoxic activity, unlike UA. Additionally, molecular docking results revealed that the UA derivatives showed a high affinity for the COX-2 active site, suggesting their anti-inflammatory effects are likely due to COX-2 inhibition. These findings suggest that compound **2** is a promising anti-inflammatory therapeutic.

**Figure 3:**
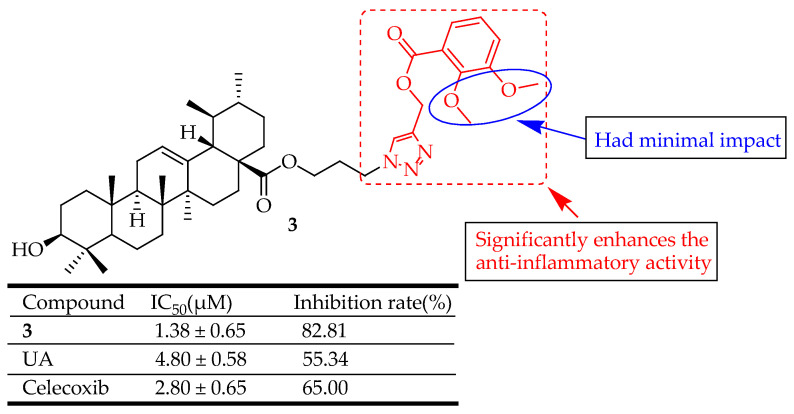
UA derivative **3** and its anti-inflammatory outcomes compared to UA and reference drug.

Zhang et al. [60] designed and synthesized three compounds by attaching 1,2,3-triazole groups to UA to explore new anti-inflammatory agents. These compounds were evaluated for anti-inflammatory effects employing an ear edema model. The most potent compound was subjected to in vitro assays for COX-2/ COX-1 inhibition. Overall, the derivatives demonstrated significant anti-inflammatory activity. Notably, compound **3** (Figure 3) showed the highest activity, with an 82.81% inhibition rate following intraperitoneal administration, surpassing celecoxib used as a positive control. Molecular docking revealed the interaction mechanism between the COX-2 enzyme and compound **3**. Further studies indicated that compound **3** had strong COX-2 inhibitory activity, with an IC_50_ value of 1.16 µM and a selectivity index (SI) of 64.66, comparable to celecoxib with an IC_50_ value of 0.93 µM and SI of 65.47. These findings show that this chemotype holds promise for developing new anti-inflammatory agents targeting COX-2. It was observed that the position and the physical and chemical properties of different substituents on the phenyl ring had little effect on the anti-inflammatory activities of these compounds. This suggests that the electronic effect of the group attached to the benzene ring was insignificant.

**Figure 4:**
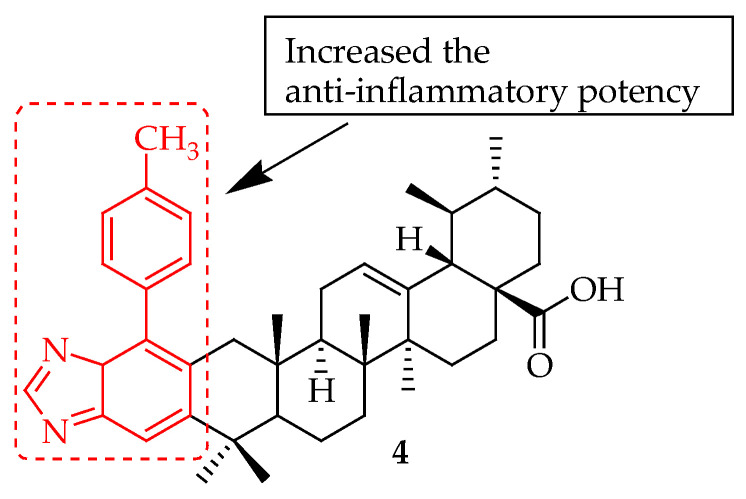
UA derivative **4** with the incorporated 1,2,4-triazolo[1,5-a]pyrimidine moiety and its corresponding effects.

The same authors [61] synthesized and screened two series of novel UA-based 1,2,4-triazolo [1,5-a]pyrimidine derivatives for their anti-inflammatory properties. They evaluated the compounds by examining how these compounds inhibit the inflammatory response induced by LPS in RAW 264.7 macrophages in vitro. The researchers examined how varying concentrations of the compounds affected the release of nitric oxide (NO) and inflammatory cytokines (i.e., TNF-α and IL-6). They also assessed the compounds’ in vitro toxicity. The findings showed that compound **4** (Figure 4) could significantly decrease the production of the inflammatory factors. A docking analysis was performed to explore how compound **4,** UA, and Celecoxib interact with the active site of the COX-2 receptor. The enzyme study conducted in vitro indicated that compound **4** achieves its anti-inflammatory effects by inhibiting COX-2. This research illustrated that incorporating a 1,2,4-triazolo[1,5-a]pyrimidine group into UA unexpectedly increased the anti-inflammatory potency of its derivatives. Previous research [20] showed a significant enhancement in the anti-inflammatory effect of UA by not modifying the carboxylic acid group (C-28). This study similarly found that certain compounds demonstrated more potent inhibition of IL-6 compared to compounds with an ethyl group added at the C-28 position.

**Figure 5:**
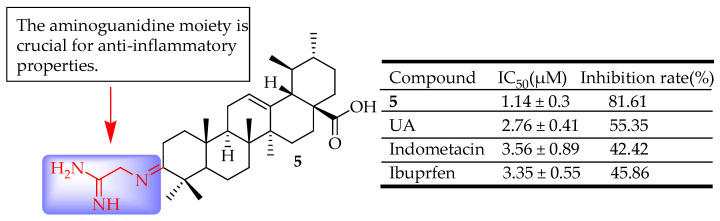
UA derivative **5** and their anti-inflammatory outcomes compared to UA and reference drugs.

Wu and colleagues [62] designed, synthesized, and evaluated three sets of UA derivatives that incorporated an aminoguanidine moiety for their antibacterial and anti-inflammatory properties. The anti-inflammatory tests revealed that a majority of the compounds demonstrated strong activity. Notably, compound **5** (Figure 5) showed the highest potency, achieving 81.61% inhibition following intraperitoneal administration. This was more effective than UA and the standard reference drugs ibuprofen and indomethacin. SAR analysis demonstrated that the aminoguanidine group was crucial for anti-inflammatory properties. These findings suggested that keeping the carboxylic group (C-28) was advantageous for maintaining this activity.

**Figure 6:**
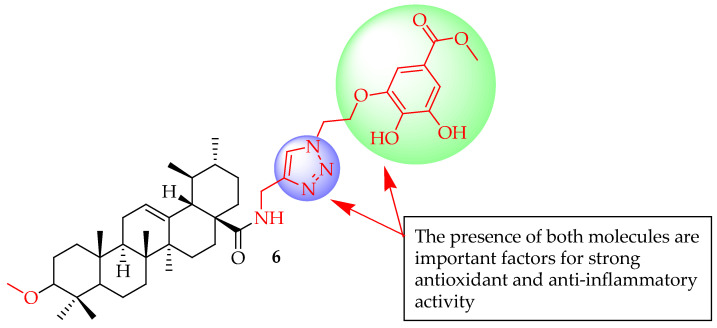
UA derivative **6** and the corresponding effect of the incorporated moieties.

Qi et al. [63] conducted a study in which they synthesized 16 new hybrids of UA. These hybrids were linked through 1,2,3-triazole to modified gallate moieties, employing CuAAC 1,3-cycloaddition reactions. In the in vitro tests, it was shown that all these derivatives were successful in reducing oxidative stress and inflammation. Significantly, compound **6** (Figure 6) effectively reduced the expression of pro-inflammatory cytokines in lipopolysaccharide (LPS)-induced RAW264.7 cells in a dose-dependent manner, notably suppressing mRNA levels of iNOS (*p* < 0.05) and COX-2 (*p* < 0.01). Compound **6**’s ability to inhibit pro-inflammatory cytokines was associated with its suppression of the LPS-induced PI3K/Akt signalling pathway. Additionally, in vivo studies using zebrafish demonstrated that compound **6** effectively reduced inflammation in the gastrointestinal tract and exhibited favourable safety profiles in cytotoxicity assessments. According to their analysis of SARS, they noted that the potent anti-inflammatory effects of these new compounds could be explained by several factors: (1) the incorporation of triazole and gallate groups as polar components along with a nonpolar triterpene structure create hybrid compounds, possessing amphiphilic properties. This improves the solubility and availability of the hybrid derivatives; (2) the strong anti-inflammatory potency of hybrid triterpene derivatives containing a triazole linker is linked to the existence of two adjacent polar substituents in the aromatic position, consistent with the research findings of Zhang et al. [62]; (3) incorporating a gallate component into the hybrid compound led to remarkable antioxidant properties, potentially accountable for the inhibition of ROS; and (4) the incorporation of shielded gallates featuring a methoxy-methylenedioxy component enhanced the antioxidant and anti-inflammatory activities. Compounds containing this methoxymethylenedioxy segment could be seen as a latent form, combining polyphenol and aldehyde attributes. Reports suggest that integrating 1,3-benzodioxole components can enhance a compound’s antioxidant, hypolipidemic effect, and anti-inflammatory (i.e., COX-2 inhibition) [64,65] properties. Hence, hybrids featuring 1,3-dioxo-lane protection may outperform derivatives with unbound phenolic OH groups in terms of antioxidant activity. The amide or ester linkage between the C-28 position of UA and the linker, along with the ether connection between the linker and the gallate element, exhibits resistance to hydrolysis, ensuring the stability of hybrid derivatives. This study offered a new reference for developing molecules for health issues related to antioxidation and anti-inflammation properties.

**Figure 7:**
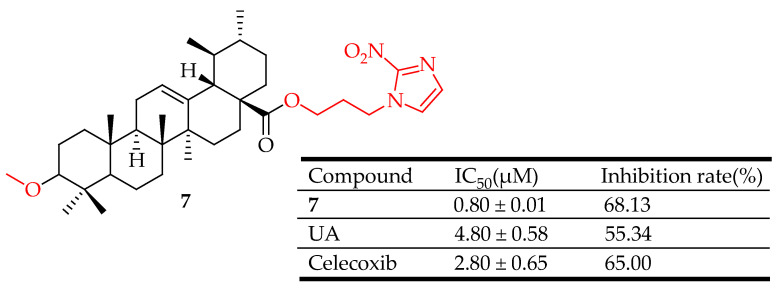
UA derivative **7** and its anti-inflammatory outcomes compared to UA and Celecoxib.

Li et al. [66] synthesized derivatives of UA and assessed their ability to inhibit HIF-1α and their anti-inflammatory properties. Compound **7** (Figure 7) demonstrated stronger inhibition of HIF-1α compared to the standard UA. In vivo tests showed that compound **7** reduced inflammation similarly to celecoxib at the same dose. Additionally, compound **7** moderately inhibited COX-2, akin to celecoxib. Overall, among the newly developed derivatives, compound **7** shows potential as a starting point for further optimization in the search for new HIF-1α inhibitors and anti-inflammatory drugs.

Overall, the reported UA derivatives exhibit notable anti-inflammatory activity by inhibiting key inflammatory mediators and pathways. These derivatives can suppress the production of pro-inflammatory cytokines and downregulate the expression of COX-2 and inducible iNOS. Additionally, some UA derivatives may enhance the activity of antioxidant enzymes, reducing oxidative stress associated with inflammation. The combined effects of these mechanisms highlight their potential application for treating inflammatory diseases or conditions, making them valuable candidates for further research and development in anti-inflammatory therapies. Below (Table 1) is a summary of the anti-inflammatory activities of UA derivatives including the method of modification, the tested Models/Assays used, and the observed effects.

### 4.2. Anticancer Activity

Currently, cancer poses a significant threat to human health in developing countries. Therefore, there is an urgent demand for new strategies and approaches to develop effective anticancer agents for cancer treatment. Currently, various studies in cancer research are focused on UA because of its efficacy throughout different stages of cancer progression and its minimal toxicity. Even though the exact mechanisms behind its effects are not well understood, many studies have demonstrated that UA can produce significant anticancer properties by regulating related factors such as apoptosis, proliferation, metastasis, and angiogenesis [67,68,69]. Indeed, UA has strong antitumor activities and its interesting molecular structure with three major active pharmacophores, such as β-hydroxy (C-3), carboxylic moiety (C-28), and alkene (C-12–C-13), makes it a distinctive compound for appropriate structural modifications to develop more innovative anticancer agents [37,59].

Structural modification of a molecule affects its receptor binding and biological activity and alters its pharmacokinetic profile and physiochemical properties [70]. To determine the significant pharmacophores of a molecular drug, a thorough understanding of its synthetic and natural analogues is required. UA has significant anticancer properties on different cancer cells without impacting healthy cells. Although the precise molecular mechanisms of UA are still unclear, scientists have demonstrated that UA exerts its anticancer effects through various pathways, including the induction of autophagy and apoptosis [35], inhibition of angiogenesis and metastasis [71], inhibition of cell invasion, arresting the cell cycle [72], and reversing drug resistance of chemotherapy [73,74].

UA appears to induce apoptosis of many cancer cell lines through various mechanisms. Apoptosis, also referred to as programmed cell death I, is a conserved intrinsic cellular mechanism playing a significant role in pathological and physiological conditions [75,76]. Cell death occurs through two distinct mechanisms: the intrinsic pathway (i.e., mitochondrial pathway) and the extrinsic pathway (i.e., receptor pathway) [77]. Chuang et al. treated hepatocellular carcinoma cells (SK-Hep-1) with different concentrations of UA (0, 10, 20, 30, 40, 50, and 60 µM) for 24, 48, or 72 h. The results showed a reduction in cell viability in a time- and dose-dependent manner, along with nuclear chromatin shrinkage, indicating that UA may induce apoptosis by inhibiting the p38MAPK- and PI3K/AKT-signaling pathways [78].

In another study by Luo et al., UA was found to trigger apoptosis in hepatoma cells (HepG2) by activating AMP-activated protein kinase (AMPK) and promoting glycogen synthase kinase 3β (GSK 3β) phosphorylation [79]. In gallbladder carcinoma cells (SGC-996 and GBC-SD), UA inhibits cell proliferation and induces S-phase cell cycle arrest and apoptosis by modulating the expression of relevant molecules. Moreover, UA administration via intraperitoneal injection reduced xenograft gallbladder tumor growth in nude mice by activating caspase-3 and caspase-9 [80].

**Figure 8:**
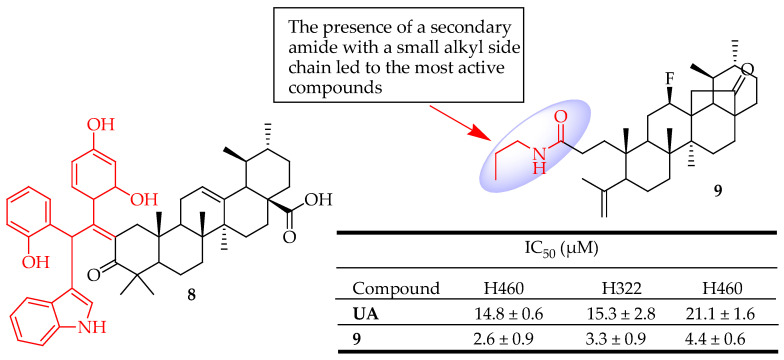
UA derivatives (**8**, **9**) and their anticancer outcomes compared to UA.

Fan et al. synthesized twelve novel UA-based hybrid compounds and evaluated them against glioma cell lines. Among the synthesized compounds, compound **8** (Figure 8) exhibited stronger inhibition of U251 cell proliferation compared to UA. Compound **8** suppressed the growth of glioma cells, triggered apoptosis, and halted cell cycle progression by down-regulating metabolic pathways [81]. Mendes et al. [82] prepared a collection of novel ring-A cleaved UA derivatives and evaluated their impact on inhibiting proliferation in non-small cell lung cancer (NSCLC) cells using both 3D and 2D culture models. The most effective compound was found to be compound **9** (Figure 8) with a secondary amine at position C-3 of a cleaved ring-A. Among the amide derivatives, those with secondary amides and bulkier side chains exhibited a significant reduction in cytotoxic activity. In contrast, secondary amides with smaller alkyl side chains resulted in the most potent compounds, with compound **9** demonstrating five times greater potency than the parent compound UA across all tested cell lines. The molecular mechanism investigation of this compound indicated the promotion of apoptosis via the activation of caspase-7/-8, along with the suppression of Bcl-2. Compound **9** also induced autophagy with elevated levels of Beclin-1 or LC3A/B-II and reduced levels of mTOR and p62.

**Figure 9:**
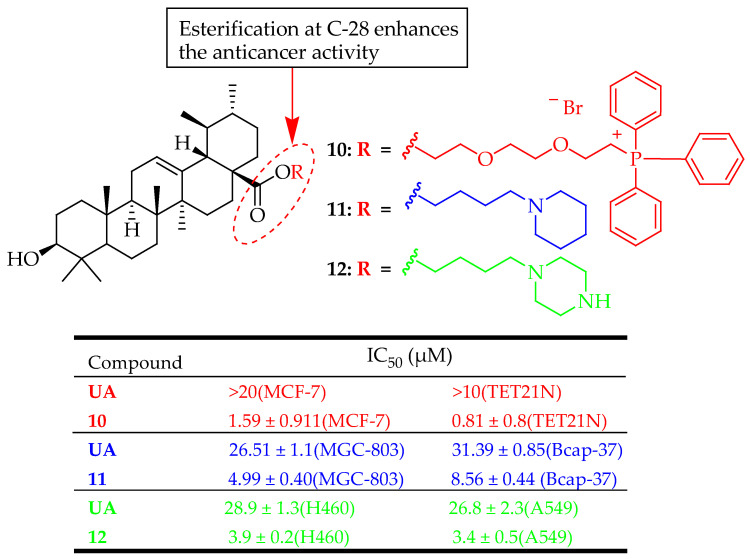
UA derivatives (**10**–**12**) and their anticancer outcomes compared to UA.

Derivative **10** (Figure 9) showed higher cytotoxicity than the parent UA in MCF-7 and TET21N cell lines with IC_50_ values of 1.59 ± 0.11 and 0.81 ± 0.08 µM, respectively. It was also significantly more effective in inducing mitochondria-dependent apoptosis, evidenced by the release of cytochrome c, activation of caspase-3, and poly(ADP-ribose)polymerase cleavage, a known caspase-3 target [83].

Liu et al. assessed the in vitro anticancer effects of compound **11** (Figure 9) on human breast cancer cells (Bcap-37) and gastric cancer cells (MGC-803) using an MTT assay. Compound **11** demonstrated a more potent inhibitory effect than UA. The mechanism of compound **11** was studied by Hoechst 33258 staining, acridine orange/ethidium bromide staining, terminal deoxynucleotidyl transferase biotin-dUTP nick-end labelling assay, and flow cytometry, which indicated that compound **11** can initiate apoptosis in MGC-803 cells, achieving an apoptosis rate of 34.59% after 36 h of treatment with 10 μM concentration [84]. The results indicate that: (1) Substituting the C28-COOH group of UA with a fatty alkyl group significantly reduced its effectiveness. (2) A notable improvement in cell growth inhibition was observed when an amino group was introduced at the C28 position. (3) Esterification of the C3-OH and C28-COOH groups with succinic anhydride and benzyl bromide, respectively, significantly enhanced the biological activity. However, introducing aromatic amines at the C-3 position resulted in a loss of activity, highlighting the importance of maintaining a polar group at the C-3 position for cytotoxic activity.

In a recent study conducted by Gou et al., a novel UA derivative compound **12** (Figure 9) exhibited a potent anticancer effect against lung cancer cells (A549 and H460 cells) than parent UA. It also revealed a stronger antiproliferation effect by inducing cell apoptosis and G0/G1 phase arrest, which is linked to the ER stress pathway, particularly the activation of the PERK/eIF2a/CHOP axis [85].

**Figure 10:**
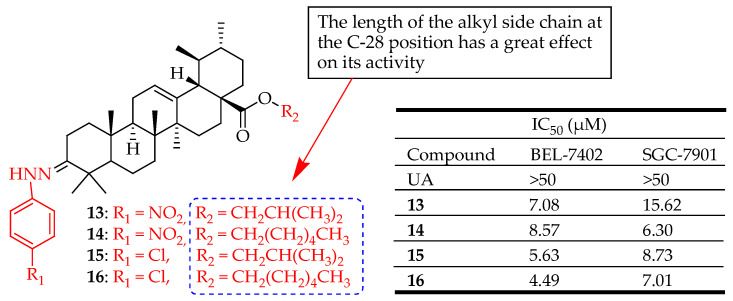
UA derivatives (**13**–**16**) and their anticancer outcomes compared to UA.

Meng et al. designed and synthesized eighteen UA derivatives and evaluated their cytotoxicity in vitro against two cancer cell lines, namely BEL7402 and SGC7901, by MTT assay. Four compounds (i.e., **13**, **14**, **15** and **16** (Figure 10)) showed a significantly higher inhibitory rate than the parent UA on both cell lines, and the interactions between the four compounds and NF-κB were also studied by docking simulations [86].

**Figure 11:**
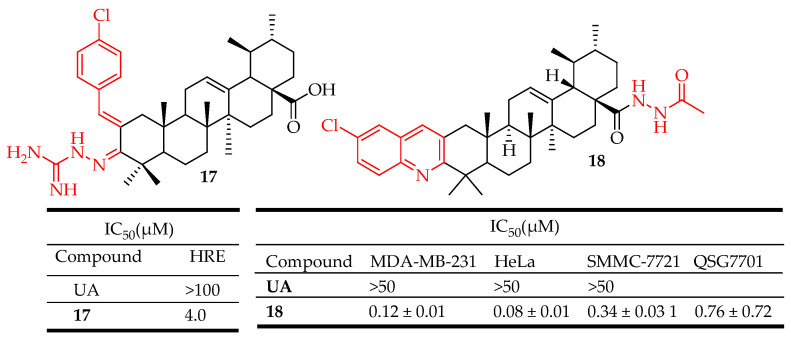
UA derivatives (**17**, **18**) and their anticancer outcomes compared to UA.

Wu et al. reported that UA derivative **17** (Figure 11) bearing an aminoguanidine moiety possesses the ability to inhibit HIF-1α transcriptional activity in low-oxygen conditions with an IC_50_ value of 4.0 µM. Compound **17** decreased HIF-1α protein expression by inhibiting its synthesis, lowered vascular endothelial growth factor production, and impeded cancer cell proliferation **[38]**. Compound **18** (Figure 11) synthesized by Jin et al. exhibited the most potent activity against three cancer cells (SMMC-7721, HeLa, and MDA-MB-231) and induced the apoptosis of cervical cancer cells (HeLa cells), halted cell cycle progression at the G0/G1 phase, decreased mitochondrial membrane potential, and elevated intracellular reactive oxygen species levels. Moreover, it suppressed MEK1 kinase activity and disrupted the Ras/Raf/MEK/ERK-signaling pathways [87].

**Figure 12:**
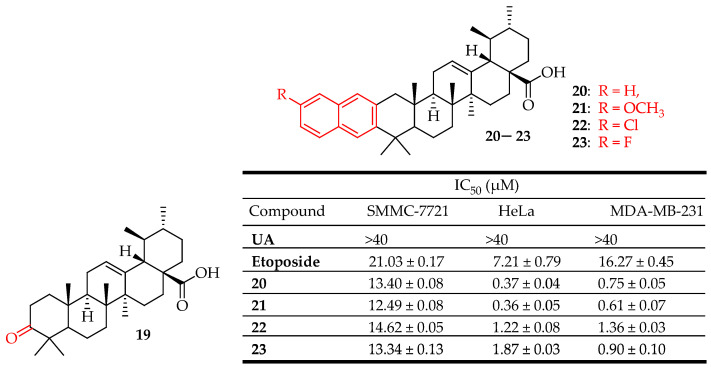
UA derivatives (**20**–**23**) and their anticancer outcomes compared to UA/model drug.

Gu et al. synthesized compounds **20**–**23** (Figure 12), which exhibited significant antitumor activities against three different human cancer cell lines (HeLa, SMMC-7721, MDA-MB-231), demonstrating greater potency than the positive control, etoposide. Compounds **20**–**23** were synthesized by dissolving UA in acetone and oxidizing it with Jones reagent, resulting in a 75% yield of 3-oxo-ursolic acid (**19**). Compound **19** was then treated with the respective o-amino benzaldehyde under a nitrogen atmosphere to produce compounds **20**–**23**, with yields ranging from 62% to 68%. Compound **21** induced apoptosis in MDA-MB-231 cell lines in a dose-dependent manner. Additionally, cell cycle analysis showed that compound **21** promoted G0/G1 phase arrest in MDA-MB-231 cell lines [88].

**Figure 13:**
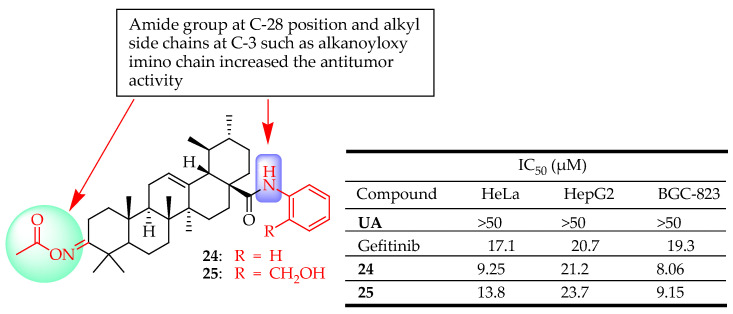
UA derivatives (**24**, **25**) and their anticancer outcomes compared to UA or a model drug.

Meng et al. synthesized eleven novel derivatives by altering positions C-2, C-3, and C-28 of UA. These derivatives were evaluated for their cytotoxicity against human cancer cells (BGC-823, HeLa and HepG2) via MTT assay. The results revealed that all the synthesized compounds had strong antiproliferative activity against HepG2, BGC-823, and HeLa cells, and the compounds that indicated the most potent activity higher than gefitinib (positive control) were derivatives **24** and **25 (Figure 13**). Converting UA into an amide group at the C-28 position enhanced the antitumor activity, as observed in compounds **24** and **25**. Additionally, alkyl side chains at the C-3 position, like alkanoyloxy imino chains, play a crucial role in inhibiting tumor cell growth [89].

**Figure 14:**
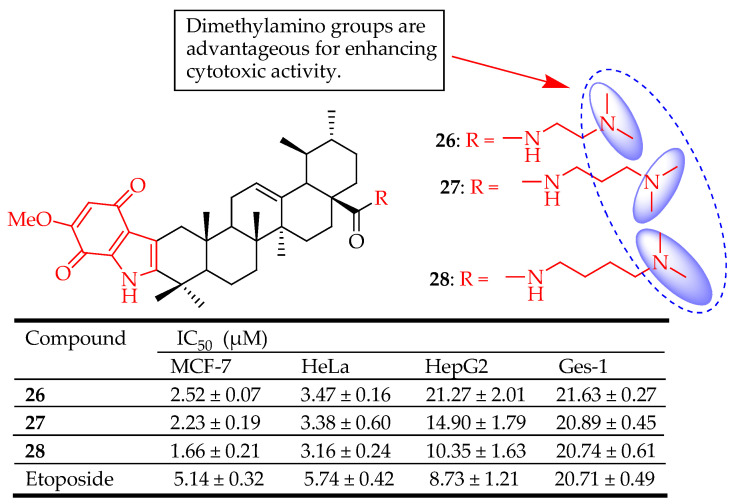
UA derivatives (**26**–**28**) and their anticancer outcomes compared to UA/reference drug.

Wang et al. [40] synthesized indolequinone derivatives of UA **26**–**28** (Figure 14) which inhibited cell migration, triggered apoptosis, and induced their cell cycle arrest of MCF-7 cells at the S phase in a concentration-dependent manner. When tested against MCF-7, HeLa, and HepG2 cells, compound **28** exhibited the best activity with IC_50_ values of 1.66, 3.16, and 10.35 µM, respectively with very low cytotoxicity against gastric mucosal cell lines (Ges-1, IC_50_ = 20.74 µM). Treating the cells with different concentrations of compound **28** raised ROS levels from 3.99% (control) to 43.23% (4 µM), suggesting that apoptosis induced by **28** was attributed to the production of ROS. The fact that **28** decreased the expression levels of p-AKT and p-mTOR indicated its ability to inhibit the P13K/AKT/mTOR-signaling pathway, an intracellular pathway important in cell cycle regulation and directly related to cell proliferation and cancer. The SAR analysis demonstrated that compounds with dimethylamino groups on the amide side chain displayed much stronger cytotoxic activities than all of the other derivatives, indicating that such moieties on the amide side chain were beneficial to their cytotoxic activity.

**Figure 15:**
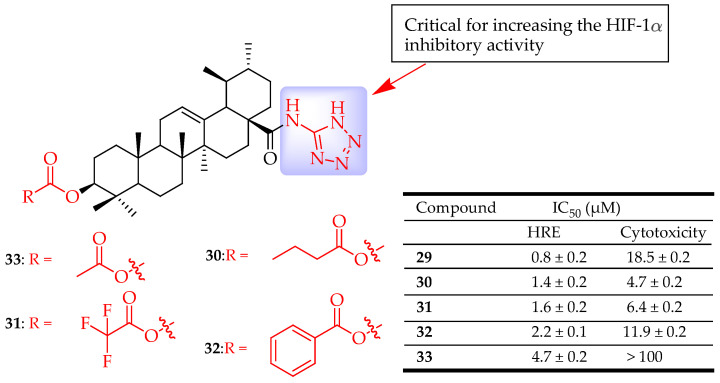
UA derivatives (**29**–**33**) and their anticancer outcomes.

Zhang et al. developed a series of new UA containing tetrazole derivatives and assessed their inhibitory activity on the hypoxia-inducible factor 1α (HIF-1α), migration, angiogenesis, and proliferation. The most potent compounds were **29** (IC_50_ = 0.8 µM), **30** (IC_50_ = 1.4 µM), **31** (IC_50_ = 1.6 µM), **32** (IC_50_ = 2.2 µM), and **33** (IC_50_ = 4.7 µM) (Figure 15). Of the compounds tested, compound **33** showed the most promising HIF-1α inhibitory activity and did not show any significant cytotoxicity at a concentration of 30 µM against a Hep3B cell line. Analysis of the SARs of the compounds showed an increase in the HIF-1α inhibitory effect upon introducing a tetrazole moiety at C-28 of UA while bulky groups at C-3 proved to decrease the HIF-1α inhibitory activity [39].

**Figure 16:**
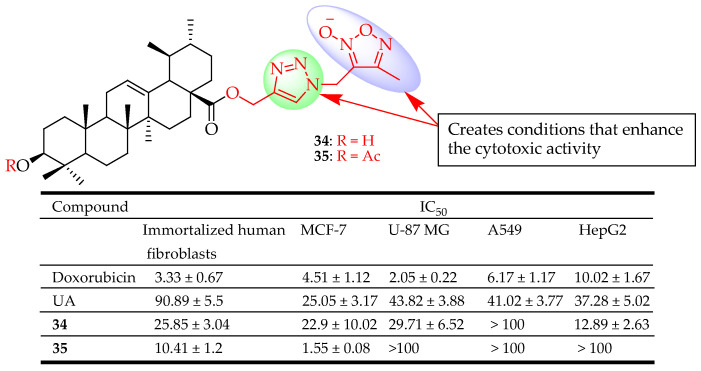
UA derivatives (**34**, **35**) and their anticancer outcomes compared to UA or a model drug.

Popov et al. synthesized novel UA derivatives with a combination of two different azole types (1,3,4-oxadiazole and 1,2,3-triazole or 1,2,5-oxadiazole and 1,2,3-triazole) at different positions of UA. These hybrid compounds were evaluated for their cytotoxicity against immortalized human fibroblasts, A549, U-87 MG, HepG2, and MCF-7 cell lines. Compounds **34** and **35** (Figure 16) showed cytotoxicity comparable to that of UA on MCF-7 cells. Compound **35** had a 3-O-acetyl group which is known to have the potential of enhancing cytotoxicity [90]. Though **35** was less active than UA against the three cell lines, it exhibited excellent cytotoxicity against MCF-7 cells with IC_50_ = 1.55 µM, even better than doxorubicin (IC_50_ = 4.51 µM). Linking heterocyclic fragments of 1,2,3-triazole and 3-(methyl)-4-methyl-1,2,5-oxadiazole-2-oxide to the C-28 position of UA creates a favorable condition for the cytotoxic activity of these hybrid derivatives [91].

**Figure 17:**
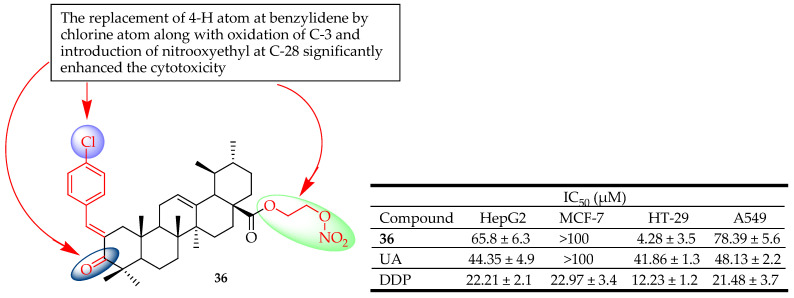
UA derivative **36** and its anticancer outcomes compared to UA or model drug.

Zhang et al. synthesized a series of NO-donating UA-benzylidine derivatives and evaluated their in vitro antitumor activity against four human cancer cell lines (HepG-2, MCF-7, HT-29, and A549). The different constituents of the benzylidene at C-2 resulted in different inhibitory activity. Compound **36** (Figure 17) was found to be the most promising candidate with IC_50_ values of 65.8, 4.28, and 78.39 µM on HepG2, HT-29, and A549 cells, respectively. The cytotoxicity of **36** against these different cancer cell lines was attributed to replacing the 4-H atom at benzylidene with chlorine, introducing nitrooxyethyl at C-28, and the oxidation of C-3. Compound **36** induced apoptosis via arrest of the cycle at the G1 phase and mitochondria-mediated pathway. The very low IC_50_ (4.28 μM) of compound **36** against HT-29 showed its potential application for the treatment of colon cancer [92].

**Figure 18:**
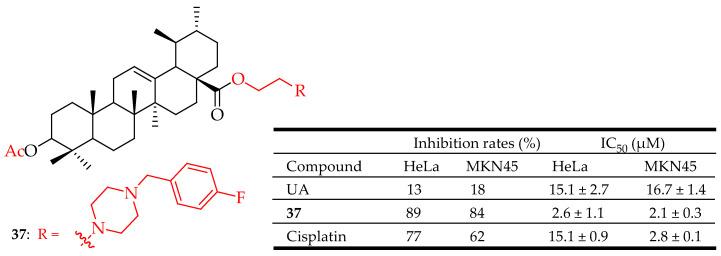
UA derivative (**37**) and its anticancer outcomes compared to UA Cisplatin.

Compound **37** (Figure 18), one of the most active compounds synthesized by Wang and colleagues, lowered the ratio of the apoptosis regulators BCL2/BAX, resulting in disrupted mitochondrial potential and triggering apoptosis. Additionally, this compound effectively suppressed the growth of Hela xenografts in nude mice. Furthermore, a SAR analysis showed that several factors significantly influenced the cytotoxicity. These factors include the acetylation of the C-3 group, the type of nitrogen heterocycle, the length of linkers between the C-28 (COOH) and nitrogen heterocycles, and various substituents on the piperazine ring [93].

**Figure 19:**
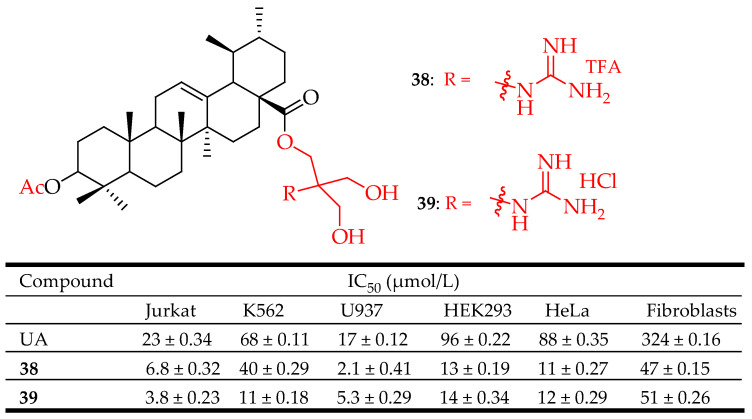
UA derivatives (**38**, **39**) and their anticancer outcomes compared to UA.

Spivak et al. evaluated the anticancer properties of their novel C-28 guanidine-functionalized UA-based derivatives. Compounds **38** and **39** (Figure 19) exhibited better anticancer activity than UA when tested against HeLa, Jurkat, Hek293, K562, and U937 cell lines. Although compound **39** showed a weaker apoptotic effect, especially on the Jurkat cell line, it showed comparable results in decreasing the number of vital Jurkat cells (6.8, 14.3, and 20.7% of early and late apoptotic cells and necrotic cells, respectively). Based on the biological evaluation, compound **39** is assumed to trigger programmed cell death, which includes apoptotic mechanisms and arresting of the cell cycle in the S-phase [94].

**Figure 20:**
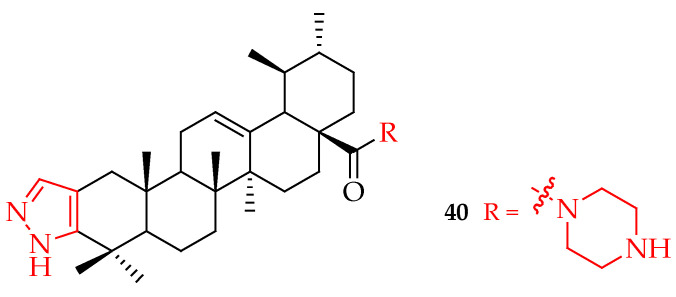
UA derivative **40** incorporated with nitrogen heterocyclic scaffolds.

Li et al. developed a novel UA derivative, having a nitrogen heterocyclic scaffold, which suppressed cell proliferation and triggered apoptosis in breast cancer cell lines. IC_50_ values for suppression of SUM149PT and HCC1937 cell viability by compound **40** (Figure 20) were 4–6 μM compared to 8-10 μM by UA on the same cell lines. Compound **40** arrested the G_0_/G_1_ cell cycle, thereby inducing suppression of cancer cell viability. Treatment of SUM149PT and HCC1937 cells with 5 μM of **40** and UA revealed that the ability of **40** to induce apoptosis was higher than that of UA. The results showed that the incorporation of piperazine and thiourea at the C-28 and C-3 positions of UA significantly inhibits breast cancer cell viability [95].

**Figure 21:**
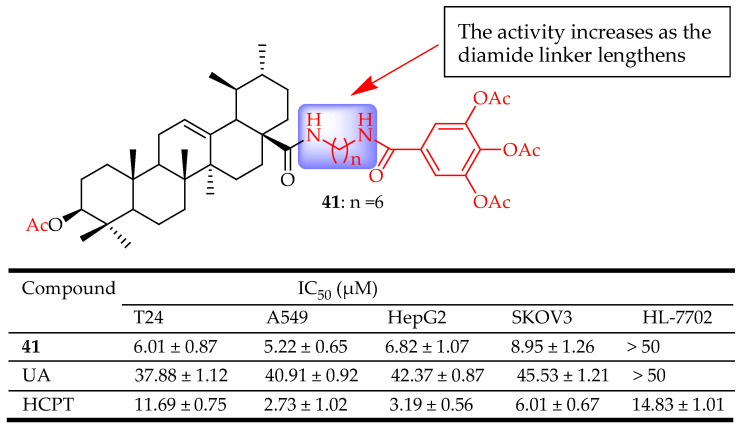
UA derivatives and their anticancer outcomes compared to UA or model drug.

Jiang et al. synthesized a series of UA derivatives having long-chain diamine gallic acid moieties as potential NF-κB inhibitors. Compound **41** (Figure 21) had the best activity against the four cell lines. The C-3 carbonyl moiety on compound **41** interacted with key residues on NF-κB through hydrogen bonding, thereby inhibiting its activity. Compound **41** inhibited the binding of NF-κB to DNA, suppressed NF-κB activation, inhibited A549 cell migration in vitro, and arrested A549 cell line at the G1 phase. The results showed the potential of the UA derivatives in inhibiting the NF-κB pathway, thus being able to suppress migration and reverse MDR in A549 lung cancer cells [36]. The SAR study reveals that diamide linkers at the C-28 position play an important role in the biological activity of the compound. Comparing the inhibitory concentrations of the pairs (with similar substitution and variation in n) indicated that the longer diamide side chain (n = 6) showed relatively enhanced activity than the shorter diamide side chain (n = 4 and 5).

**Figure 22:**
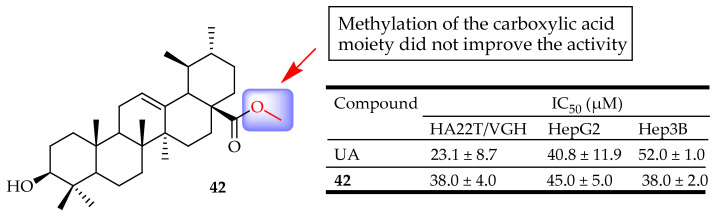
UA derivative **42** and their anticancer outcomes compared to UA.

Fontana et al. evaluated the involvement of NF-κB in the cytotoxicity of UA derivatives towards the cell lines HepG2, Hep3B, and HA22T/VGH of hepatocellular carcinoma. Methylation of the carboxylic acid moiety did not improve the activity of the compounds while oxidation of C-3 resulted in the loss of activity. Compound **42** (Figure 22) showed inhibitory effects on NF-κB comparable to the ones of UA against the selected cell lines, showing its potent cytotoxicity towards hepatocellular carcinoma [33].

UA derivatives have shown significant anticancer activity through various mechanisms, including induction of apoptosis, inhibition of tumor cell proliferation, and modulation of signaling pathways, such as NF-κB, which is associated with cancer progression. These derivatives can disrupt the cell cycle, promote the generation of ROS, and enhance the expression of pro-apoptotic proteins while downregulating anti-apoptotic factors. Additionally, these derivatives have demonstrated the capability to inhibit angiogenesis by affecting tumor microenvironment interactions and inflammatory responses. Overall, the structural modification of UA has led to the development of derivatives with superior anticancer properties, making them promising candidates for further preclinical and clinical development in cancer therapy. Table 2 summarizes the anticancer activities of the reported UA derivatives, including the method of modification, the tested cancer cell lines, and the observed effects. Subsequent research is still needed to explore the in-depth structure-activity relationships and the antitumor mechanism of these derivatives.

### 4.3. Antimicrobial

The extensive use and misuse of antibiotics give rise to microbial drug resistance, which is a serious challenge. Currently, several therapeutic compounds are being developed, however, the issue of drug resistance is increasing. Drug resistance is one of the world’s most serious health issues [96]. Bacterial infections caused by drug-resistant pathogens are much worse compared to antibiotic-susceptible ones [97]. The only way to combat these bacterial infections is to develop novel antibiotics or combinations of two or more antibiotics with different modes of action. Previous research suggests that UA and its analogues may inhibit bacterial growth by disrupting metabolic pathways [9]. Other research has found that lipophilic molecules like pentacyclic triterpenoids can disrupt membrane stability and halt cell growth [98]. Triterpenoids are thought to inhibit bacterial efflux pumps, DNA synthesis, and macromolecular synthesis in Gram-positive bacteria [98,99,100]. Due to the lack of data, the exact antibacterial mechanism for triterpenoids is not yet known, but it was demonstrated that the lack of β-hydroxyl group in position C-3 of betulinic acid derivatives did not exert antibacterial activities against *Staphylococcus aureus* and *Escherichia coli* [101]. Triterpenoids’ biological activity is enhanced by modifying them at the C-28 COOH position via amination and esterification [102,103,104,105]. It has been brought to light that triterpenoids and their derivatives may be useful weapons to solve the issue of multidrug resistance and reduce the side effects of antibiotics [101].

#### 4.3.1. Antibacterial Activity

UA and its derivatives present a multifaceted antibacterial approach, targeting membrane integrity, biofilm formation, enzyme activity, efflux mechanisms, oxidative stress, and metabolic pathways. These diverse mechanisms not only reduce bacterial growth and survival but also enhance the effects of conventional antibiotics, making UA a promising candidate in the fight against bacterial infections, especially those involving resistant strains. Additionally, studies have explored the use of UA in conjunction with antibiotics as a promising alternative for treating bacterial infections. For instance, Wojnicz et al. [106] investigated the combination of UA and ciprofloxacin, which is used to treat recurrent urinary tract infections caused by *E. coli*. Their findings revealed an improved antibiofilm efficacy against *E. coli,* potentially due to the acidic nature of UA.

**Figure 23:**
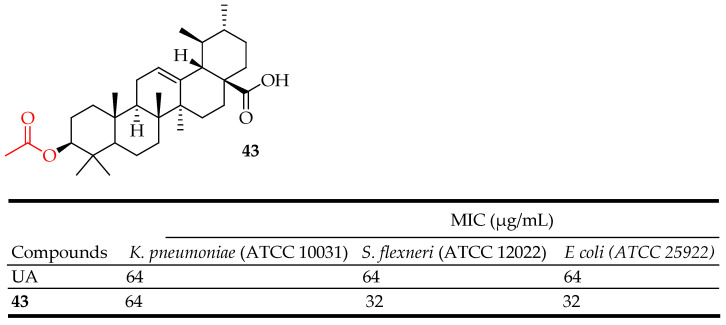
UA derivative **43** and its antibacterial outcomes compared to UA.

UA (32 μg/mL) also reportedly synergizes with colistin when used to treat clinical *Klebsiella pneumoniae* BC936 and *E. coli* U3790 isolates [107]. Cunha et al. reported that UA isolated from *Miconia ligustroides* was active against *Bacillus cereus* with a MIC value of 20 μg/mL. The ester methylation and acetylation of UA improved the inhibitory activity against *Streptococcus pneumonia* [108]. Furthermore, UA derivatives demonstrated broad-spectrum antibacterial activities against both Gram-negative and Gram-positive bacteria. Do Nascimento and colleagues [109] synthesized two semi-synthetic compounds by modifying the UA structure at C-3. They investigated how UA and its derivatives affected the susceptibility of certain bacterial pathogens to aminoglycoside antibiotics, including neomycin, amikacin, kanamycin, and gentamicin. The most notable synergistic effect was observed with derivative 3β-formyloxy-urs-12-en-28-oic acid (**43**) (Figure 23) at a concentration of 32 μg/mL against *Shigella flexneri* and *E. coli*, a multidrug-resistant clinical isolate from sputum.

**Figure 24:**
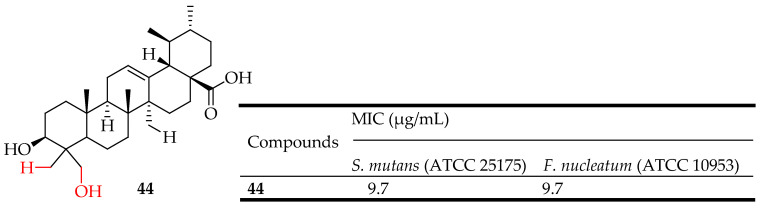
UA derivative **44** and its antibacterial outcomes compared to UA.

Zhao et al. extracted UA from the *Ilex hainanensis* Merr. leaves and synthesized seven UA-based derivatives. They assessed their antibacterial efficacy by measuring their MIC against both Gram-positive (*Streptococcus mutans* ATCC 25175) and Gram-negative (*Fusobacterium nucleatum* ATCC 10953) bacterial strains. Among the synthesized derivatives, compound **44** (Figure 24) demonstrated a notable effect against *S. mutans*, with a MIC of 9.7 μg/mL, but showed minimal antibacterial activity against *F. nucleatum* [110]. Oloyede et al. investigated the antibacterial properties of UA by examining how reactive oxygen species and oxidative stress contribute to its effectiveness against *Pseudomonas aeruginosa*, *S. aureus*, and *E. coli.* They found that the viability of bacteria treated with UA decreased over time with MIC of 256 mg/mL for *E. coli and P. aeruginosa,* and 64 mg/mL for *S. aureus*. Interestingly, when bacteria were treated with UA in the presence of 2,20-bipyrydyl, cell viability increased. The study also observed a significant (*p* < 0.05) increase in superoxide anion production in bacteria treated with UA. Furthermore, the ratio of NAD+/NADH significantly increased (*p* < 0.05) in these bacteria. Moreover, UA treatment led to a significant decrease in reduced glutathione levels and an increase in glutathione disulphide, malondialdehyde, and fragmented DNA in *E. coli*, *P. aeruginosa*, and *S. aureus*. These findings strongly indicate that UA shows promise as an effective antibacterial agent [27].

Previous studies indicate that several triterpenoids demonstrate synergistic effects with various classes of antibiotics, highlighting the potential of plant-derived compounds to enhance antibiotic efficacy against multidrug-resistant pathogens. To elucidate the mechanism by which triterpenoids combat these resistant bacteria. Wang and colleagues analyzed the SAR of UA against *S. aureus*. They investigated how UA could affect both bacterial and mammalian membranes. They employed 2D proteomic analysis to study how methicillin-resistant *S. aureus* responds at the proteomic level to treatment with UA [111]. Another study by Pandey et al. explored the antibacterial effect of UA derived from *Ocimum sanctum* against *E. coli*. They observed dose-dependent enhancement in its activity at concentrations of 15, 20, and 25 mg/mL. At these concentrations, the effectiveness of UA showed comparable efficacy to the standard drug albendazole. The study utilized both disk diffusion and well diffusion methods to screen UA’s antibacterial efficacy. In vitro antimicrobial tests indicated that UA exhibited promising antibacterial properties [112]. Furthermore, Park et al. [113] explored the antibacterial potential of three distinct saponin triterpenoids, including UA, employing diverse methodologies. They employed quantitative real-time PCR (qPCR) and microarray analysis to investigate the expression of genes linked to key metabolic pathways in *S. mutans* UA159 after incubation with UA. An oligonucleotide array containing 5363 probes was designed to examine 1928 of the 1963 genes in the *S. mutans* UA159 genome. Genes exhibiting a 2-fold change in expression due to the treatment were identified, and qPCR was used to analyze a selection of target genes involved in central metabolism. The gene expression patterns of UA-treated cells, as revealed by microarray analysis, indicated alterations in the antimicrobial mechanism. This finding suggests that UA-treated cells exhibit a promising antimicrobial mechanism worthy of further investigation.

Qian et al. [114] evaluated the antimicrobial mechanism of UA against carbapenem-resistant *Klebsiella pneumoniae* (CRKP). Their findings indicate that UA is effective against CRKP at a minimum inhibitory concentration (MIC) of 0.8 mg/mL. UA was found to compromise the integrity of CRKP cell membranes, inhibit biofilm formation and the expression of biofilm-related genes, and inactivate CRKP cells within biofilms. This study investigated the antibacterial properties and mechanisms of UA against CRKP, using the agar dilution method to determine UA’s MIC. To assess UA’s impact on the cell membrane, researchers monitored changes in intracellular pH, ATP content, and cell membrane potential. The results suggest that UA could be a promising treatment for multidrug-resistant *K. pneumoniae* infections when used alongside other antibiotics.

**Figure 25:**
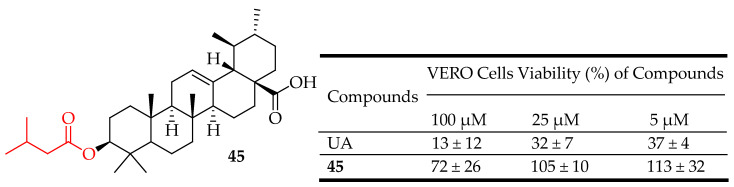
UA derivative **45** and its antibacterial outcomes compared to UA.

To find treatments that inhibit the development of biofilms, numerous researchers have reported intriguing results. Nine derivatives of UA were evaluated for their in vitro antibacterial efficacy against both planktonic and biofilm cells of gram-positive pathogens like *Enterococcus faecalis*, *S. epidermidis,* and *S. aureus*. The researchers assessed the antibiofilm properties of these analogues, including UA, using the crystal violet method, and measured their antibacterial effectiveness through absorbance (OD600) at different concentrations (5, 25, and 100 µM). Additionally, they evaluated the in vitro cytotoxicity of similar molecules on African green monkey (VERO) cells using the MTT assay at the same concentrations. They observed that a C-3 substitution in the UA chemical structure enhanced antibiofilm activity. Notably, among all the promising UA analogues, compound **45** (Figure 25) emerged as the most active molecule with minimal or no toxic effects against mammalian cells [115].

The few UA derivatives exhibit significant antibacterial activity against a wide range of pathogenic bacteria, including both Gram-positive and Gram-negative strains. Studies have shown that these compounds can effectively inhibit the growth of antibiotic-resistant strains, making them promising candidates for addressing the growing issue of antibiotic resistance. Additionally, UA derivatives may enhance the efficacy of conventional antibiotics when used in combination, potentially leading to synergistic effects. Overall, the diverse antibacterial properties of UA derivatives underscore their potential as effective natural agents for treating bacterial infections. The summary of the antibacterial activity of UA derivatives is shown in Table 3 below, including the method of modification, the tested bacterial strains, and the observed effects.

#### 4.3.2. Antiviral Activity

UA and its derivatives have shown promising antiviral activities against a variety of viruses such as HIV, influenza, etc. UA can inhibit Hepatitis B Virus (HBV) replication and suppress HBx-mediated pathways, which are crucial for the virus’s lifecycle. This includes the suppression of RhoA activation, beclin-1 promoter activation, and autophagy induction, as well as reversing HBx-induced drug resistance [117].

##### Human Immunodeficiency Virus (HIV)

**Figure 26:**
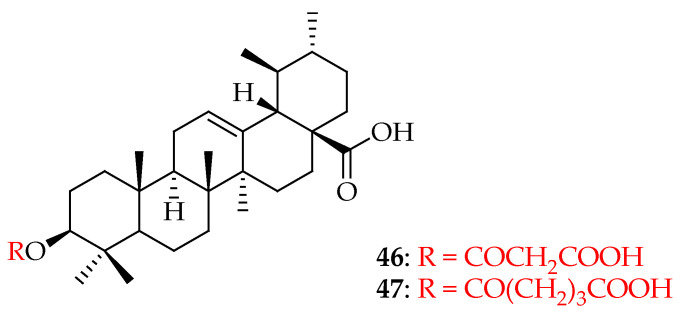
UA derivatives (**46**, **47**) extracted from stems of *Cynomorium songaricum*.

It is estimated that approximately 36.7 million people worldwide were living with AIDS at the end of 2017. Year after year, morbidity and mortality rates have risen dramatically. More than thirty drugs targeting various stages of the HIV viral life cycle have been approved so far for the management of HIV/AIDS. However, serious issues such as the emergence of extensively drug-resistant bacteria and negative side effects remain [118]. Hence, there is a necessity to develop anti-HIV/AIDS drugs that exhibit potent therapeutic effects and favorable pharmacokinetic profiles, with minimal or no adverse effects. Several pentacyclic triterpenoids and their saponin derivatives have shown anti-HIV activity [119]. UA and its hydrogen malonate derivative **46** (Figure 26), extracted from the stems of *Cynomorium songaricum*, exhibit HIV-1 protease inhibition with EC_50_ values in the micromolar range. Additionally, the glutaryl hemiester compound **47** (Figure 26) of UA has demonstrated anti-HIV-1 protease activity at a concentration of 4 Μm [120].

**Figure 27:**
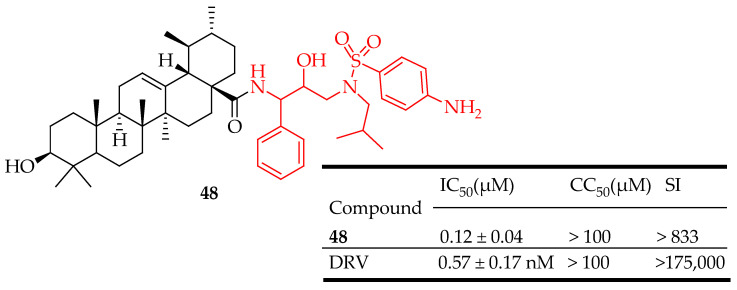
UA derivative **48** and its antiviral outcomes compared to model drug.

Zhu et al. [118], developed a series of derivatives of UA, utilizing them as P2 ligands, along with phenyl sulfonamide as P2′ ligands, to investigate their SAR as inhibitors of HIV-1 protease. The results indicated that these derivatives exhibited micromolar inhibitory activity. Specifically, compound **48** (Figure 27) demonstrated potent inhibition of HIV-1 protease with IC_50_ = 0.12 μM), which was reported to be 67 times more effective than the parent compound UA (IC_50_ = 8.0 μM). These findings suggest that P2 ligands may not effectively complement the residues of the protease S2 subsite. Further research is recommended to explore pentacyclic terpenoid fragments, like those in UA, as potentially superior inhibitors of HIV targets beyond HIV-1.

##### Influenza Virus

**Figure 28:**
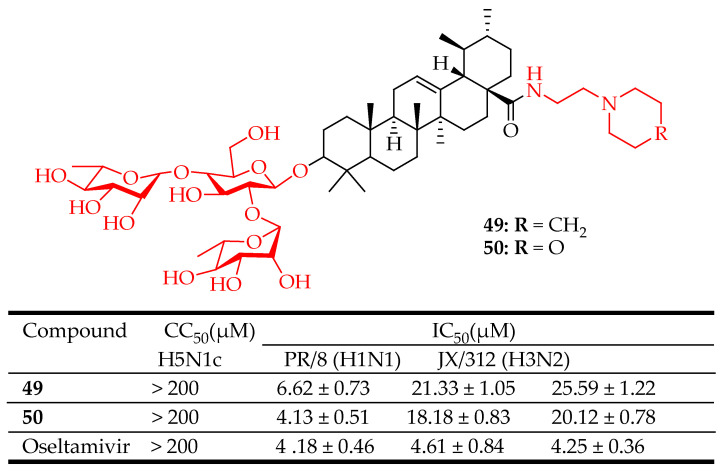
UA derivatives and their antiviral outcomes compared to reference drug.

Several viral changes have led to the formation of consistent strains of influenza A viruses (IAVs) within vulnerable human populations: (H1N1, H1N2, H2N2, and H3N2) [121]. Because of genetic changes caused by antigenic shifts and occasional antigenic drifts, IAVs exhibit high pathogenicity and are responsible for annual epidemics and occasional global pandemics of respiratory diseases. The highly pathogenic avian influenza (HPAI) H5N1 virus, specifically, has posed significant health and economic risks worldwide. Interestingly, certain protease inhibitors, such as pentacyclic triterpenoids, have demonstrated effective inhibition of IAVs through straightforward modifications of established natural pentacyclic triterpenoids or through innovative discovery methods [122]. Li et al. [122] synthesized derivatives of UA (**49** and **50**) (Figure 28) in their search for an effective inhibitor against IAVs. Compounds **49** and **50** were evaluated for their ability to inhibit the H5N1 virus and two other strains using a cytopathic effect reduction assay in A549 cells. Additionally, compounds **49** and **50** were subjected to an MTT assay on A549 cells to determine their cytotoxic effects relative to their antiviral activity. The results indicated that these derivatives demonstrated effective inhibition against the H5N1 virus at micromolar concentrations, although their antiviral potency was comparable to or slightly less than that of the standard drug (oseltamivir). Compound **50** was found to interfere with viral hemagglutinin, thereby preventing infection by H1, H3, and H5 types of influenza A viruses. Furthermore, the antiviral efficacy observed in experimental assays correlated well with docking studies, suggesting that compound **50** merits further optimization and development as a promising new lead compound.

**Figure 29:**
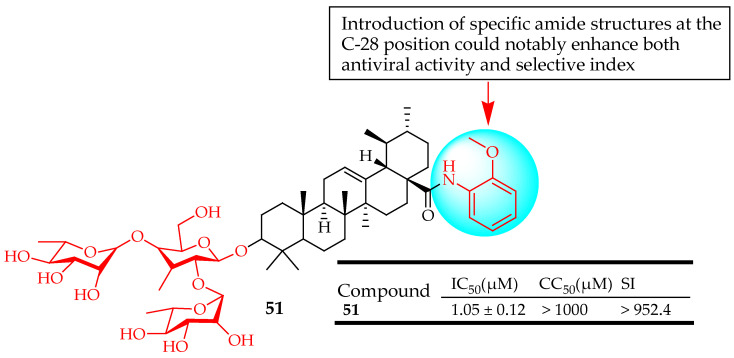
UA derivative **51** and its antiviral outcomes.

Liao et al. [123] prepared a range of pentacyclic triterpene saponins modified at C-28 through conjugation with diverse amide derivatives. They assessed the antiviral properties of these compounds against the influenza A/Duck/Guangdong/99 virus (H5N1) using MDCK cells. Among these derivatives, compound **51** (Figure 29) significantly inhibited influenza A virus replication in a dose-dependent manner, aligning well with cytopathic effect reduction results. The SAR analysis indicated that introducing specific amide structures at the COOH position of UA could notably enhance both antiviral activity and selective index. The study highlighted that attaching a methoxy group or a Cl atom to the phenyl ring at the ortho- or para-position was essential for improving inhibitory activity. Mechanism studies showed that these triterpenoids could bind tightly to the viral envelope hemagglutinin, blocking the virus’s attachment to host cells, consistent with docking studies.

**Figure 30:**
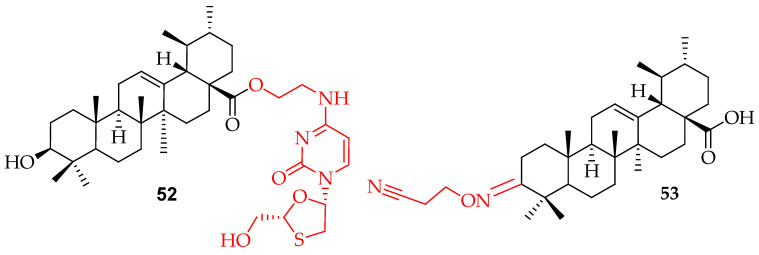
UA derivatives **52**, **53** linked with lamivudine at C-28 and cyanoethyloximino at C-3.

A new codrug, referred to as compound **52** in Figure 30, which combines lamivudine and UA through an ester bond, had the dual action of anti-hepatitis B virus activity and hepatoprotective effects against acute liver injury [124]. The antiviral screening of the cyanoethyloximino derivative (**53**) of UA against human papillomavirus type 11 showed a selectivity index of 30, with no observed cellular cytotoxicity [14].

It has been observed that UA derivatives exhibit significant antiviral activity against a range of viral pathogens, demonstrating their potential application as therapeutic agents for treating viral infections. These compounds exert their antiviral effects through various mechanisms, including the inhibition of viral entry into host cells, disruption of viral replication, and modulation of the host immune response. Studies have shown that UA derivatives can interfere with viral enzymes, such as proteases. Overall, the diverse antiviral mechanisms of UA derivatives highlight their potential use for the development of effective therapeutics against various viral diseases. Table 4 below summarizes the antiviral activity of various UA derivatives, including the method of modification, the tested viruses, and the observed effects.

#### 4.3.3. Antioxidant Properties

UA acts as a potent antioxidant by scavenging reactive oxygen species (ROS) and upregulating endogenous antioxidant enzymes [111]. This helps protect cells from oxidative stress-induced damage, which is implicated in various chronic diseases, including neurodegenerative disorders. It can act effectively as a radical scavenger, a chain-breaking antioxidant, or a chelator of metals that generate radicals. It is widely recognized that many commonly used drugs, such as anticancer agents, non-steroidal anti-inflammatory drugs, antiretroviral agents, antipsychotics, and analgesics, can induce harmful free-radical toxicity. The metabolism of these drugs can produce reactive intermediates that directly reduce molecular oxygen, leading to the formation of reactive oxygen species [125].

**Figure 31:**
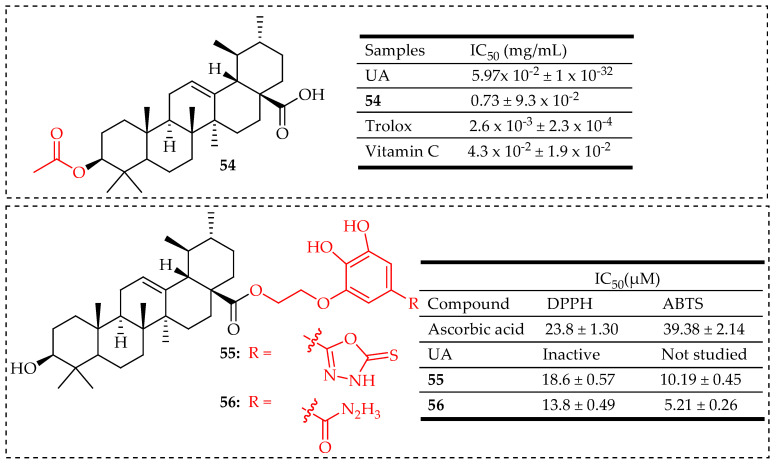
UA derivatives (**54**–**56**) and their antioxidant outcomes compared to UA/reference drugs.

In a study by Do Nascimento et al. [109] compound **54** (Figure 31) demonstrated antioxidant properties by inhibiting DPPH. They effectively scavenged the DPPH radical, with IC_50_ values of 5.97 × 10^−2^ ± 1 × 10^−3^ mg/mL and 0.73 ± 9.3 × 10^−2^ mg/mL, respectively. In comparison, Trolox and Vitamin C, used as positive controls in this study, had IC_50_ values of 2.6 × 10^−3^ ± 2.3 × 10^−4^ mg/mL and 4.3 × 10^−2^ ± 1.9 × 10^−2^ mg/mL. Popov et al. [116] developed new hybrid derivatives of UA (**55** and **56**) (Figure 31), incorporating hydrazide and 1,3,4-oxadiazole groups, which demonstrated significant antioxidant activity.

The reported UA derivatives possess potent antioxidant properties, which are attributed to their ability to scavenge free radicals and reduce oxidative stress in biological systems. Table 5 summarizes the antioxidant activity of UA derivatives, including the method of modification, the antioxidant assays, and the observed effects.

#### 4.3.4. Antidiabetic Activity

UA and its derivatives hold significant promise as antidiabetic agents due to their multifaceted mechanisms of action, including improving insulin sensitivity, reducing gluconeogenesis, anti-inflammatory and antioxidant effects, and activating AMPK [126]. In recent years, researchers have investigated structural modifications of UA to develop new derivatives with improved antidiabetic properties.

**Figure 32:**
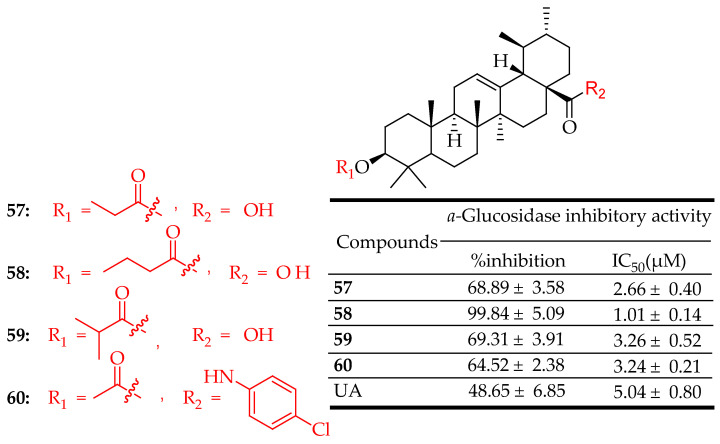
UA derivatives (**57**–**60**) and their antidiabetic outcomes compared to UA.

In a research conducted by Wu et al. [28] various derivatives of UA displayed notable inhibitory effects, particularly compounds **57**–**60** (Figure 32), with IC_50_ values of 2.66 ± 0.84, 1.01 ± 0.44, 3.26 ± 0.22, and 3.24 ± 0.21 μM, respectively. These compounds exhibited greater potency against α-glucosidase compared to acarbose, the positive control. To understand their inhibitory mechanisms, kinetic studies were conducted. Compound **57** was identified as a non-competitive inhibitor with an inhibition constant (Ki) of 2.67 ± 0.19 μM. On the other hand, compounds **58**–**60** were found to be mixed-type inhibitors through kinetic inhibition studies. Furthermore, the practical pharmacological effects of synthesized compounds **57** and **58** were demonstrated by their ability to lower postprandial blood glucose levels in normal Kunming mice.

**Figure 33:**
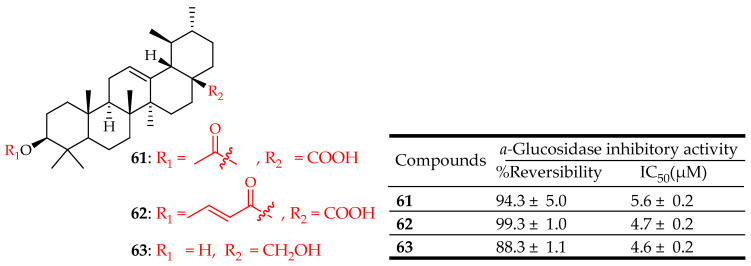
UA derivatives (**61**–**63**) and their antidiabetic outcomes.

Guzman Avila et al. [29] synthesized seven derivatives of UA. Among them, compounds **61**, **62**, and **63** (Figure 33) showed substantial inhibitory effects on the PTP-1B enzyme in a reversible manner. Compound **63** exhibited the highest activity, demonstrating significant effects both in vitro and in vivo. Furthermore, acetyl and crotonyl esters were identified as the most potent derivatives in experimental setups. Molecular docking analysis indicated that acetyl and crotonyl derivatives exhibited better binding scores compared to the parent compound, UA.

**Figure 34:**
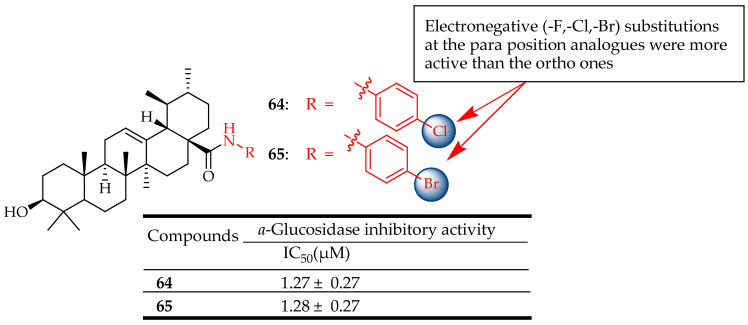
UA derivatives (**64**, **65**) and their antidiabetic outcomes.

The findings from Wu et al.’s study indicated significant inhibitory activity among most analogues of UA, particularly analogues **64** and **65** (Figure 34), which displayed IC_50_ values of 1.27 ± 0.27 μM and 1.28 ± 0.27 μM, respectively. These values were lower compared to other synthesized analogues and the control. Among these, analogues with electronegative (-F, -Cl, -Br) substitutions at the para position were more active than those with substitutions at the ortho position, particularly analogues **64** and **65**. 2D-QSAR and molecular docking analysis were conducted to demonstrate that the C-3 position could interact with the hydrophobic region of the active pocket, forming hydrogen bonds to enhance the binding affinity of the ligand to the homology-modelling protein. Consequently, these findings offer insights into the correlation between binding mechanisms and bioactivity, aiding in the design of improved inhibitors derived from UA analogues [127].

**Figure 35:**
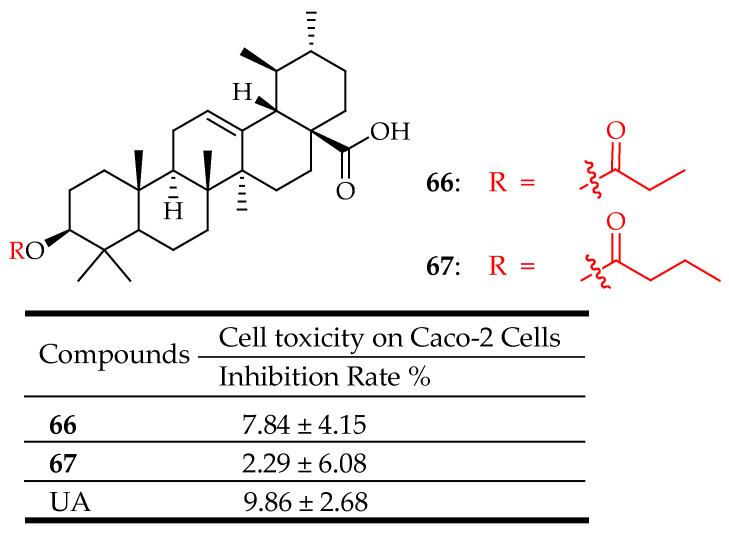
UA derivative (**66**, **67**) and their antidiabetic outcomes compared to UA.

In another study conducted by the same researchers [128], compounds **66** and **67** (Figure 35) exhibited significant inhibition of 2-NBDG uptake under both sodium-dependent and sodium-independent conditions. This inhibition was achieved by reducing the expression of SGLT-1 and GLUT-2 in the Caco-2 cell model. Subsequent in vivo studies demonstrated that compound **66** notably alleviated hyperglycemia by enhancing serum insulin levels, total protein, and albumin. Moreover, it effectively normalized fasting blood glucose levels, body weight, and food intake, bringing them closer to those of healthy rats. Compounds **66** and **67** also demonstrated hypolipidemic effects by reducing total cholesterol and triglyceride levels. Additionally, compound **66** exhibited antioxidant properties, as evidenced by increased levels of glutathione and superoxide dismutase, along with decreased levels of malondialdehyde in the liver and kidneys of diabetic rats.

**Figure 36:**
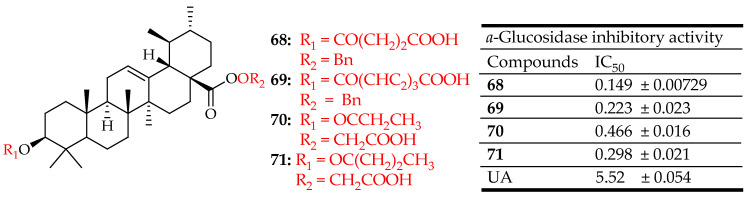
UA derivatives (**68**–**71**) and their antidiabetic outcomes compared to UA.

Huang et al. [129] developed and synthesized a novel series of UA derivatives aimed at potentially serving as anti-diabetic agents through the inhibition of α-glucosidase. Their findings from half-maximal inhibitory concentration assays indicated that all tested compounds exhibited greater potency against α-glucosidase compared to acarbose. Notably, compounds **68**–**71** (Figure 36), which featured specific long hydrophilic groups at the C-3 or C-8 positions, demonstrated inhibitory activity ranging from twelve to thirty-seven times higher than the parent compound UA. However, compounds bearing free carboxyl groups at both the C-3 and C-28 positions showed reduced enzyme inhibition activity. Additionally, UA derivatives conjugated with hydrophobic groups displayed diminished inhibitory effects against Baker’s yeast α-glucosidase. Mechanistically, compounds **68** and **69** were found to inhibit α-glucosidase through a mixed-type inhibition, while compounds **70** and **71** exhibited a non-competitive inhibition mechanism. Moreover, the correlation between IC_50_ values and binding free energies indicated that docking simulations provided valuable predictive insights. These results suggest that UA derivatives modified with polar and hydrophilic moieties could represent a promising new class of compounds worthy of further investigation in animal studies or clinical trials as potential anti-diabetic agents.

UA derivatives demonstrate promising antidiabetic activity through several mechanisms that enhance glucose metabolism and improve insulin sensitivity. These compounds have been shown to increase glucose uptake in muscle and adipose tissues by upregulating glucose transporter proteins, such as SGLT-1 and GLUT-2. Additionally, UA derivatives can inhibit α-glucosidase activity, and the PTP-1B enzymes in a reversible manner. Overall, the multifaceted actions of UA derivatives position them as valuable candidates for the development of novel therapeutic agents in diabetes management. Table 6 below summarizes the antidiabetic activity of various UA derivatives, including the method of modification, the tested models or assays, and the observed effects.

#### 4.3.5. Conclusions

The synthesis of UA derivatives is currently of great interest to medicinal and organic chemists because of its strong pharmacological effects. The structural modification of UA significantly enhances its biological activities. Ongoing research in this field continues to uncover new derivatives with promising pharmacological properties aiding in the design of more effective therapeutic agents. Most identified derivatives of UA have shown greater potency than both standard drugs and the original compound, UA.

After reviewing the wide range of pharmacological activities of UA derivatives, it was noted that the anticancer properties of UA derivatives have garnered significant attention over the past decade, as most of the derivatives discussed in this study originate from the development of anticancer drugs. UA has been observed to impact numerous targets throughout different stages of cancer progression, including apoptosis, proliferation, angiogenesis, and metastasis. Compounds **8**–**42** (Figure 8, Figure 9, Figure 10, Figure 11, Figure 12, Figure 13, Figure 14, Figure 15, Figure 16, Figure 17, Figure 18, Figure 19, Figure 20, Figure 21 and Figure 22) are representative UA-based compounds that demonstrate enhanced anticancer effects in comparison to either UA itself or the model drug.

In anti-inflammatory terms, UA derivatives suppress the activation of NF-κB, a transcription factor that plays a crucial role in regulating the expression of various pro-inflammatory cytokines and enzymes. They can downregulate cyclooxygenase-2 (COX-2) and inducible nitric oxide synthase (iNOS), enzymes involved in the production of inflammatory mediators. Compounds **2**–**7** (Figure 2, Figure 3, Figure 4, Figure 5, Figure 6 and Figure 7) exemplify UA derivatives that exhibit superior anti-inflammatory effects compared to UA and the standard drugs used as controls.

The antidiabetic activities of the UA derivatives include the inhibition of α-glucosidase, an enzyme involved in carbohydrate digestion, thereby reducing postprandial blood glucose spikes. They enhance insulin-signaling pathways, improving glucose uptake in peripheral tissues. Compounds **57**–**71** (Figure 32, Figure 33, Figure 34, Figure 35 and Figure 36) are representative UA-based compounds with enhanced antidiabetic activity.

UA exhibits significant antibacterial activity against Gram-negative and Gram-positive bacteria such as S. aureus, including methicillin-resistant *S. aureus* (MRSA). It disrupts bacterial cell walls and inhibits bacterial enzymes essential for survival and replication. Compounds **43**–**45** (Figure 23, Figure 24 and Figure 25) are representative UA-based compounds with enhanced antibacterial activity.

In the field of antivirals, the derivatives of UA showed strong antiviral activity, mainly against HIV, influenza, and herpes. UA derivatives have demonstrated activity against HIV by inhibiting key enzymes such as reverse transcriptase and protease, essential for viral replication. UA has been shown to inhibit the replication of influenza viruses. It interferes with the viral entry into host cells and the replication of viral RNA. Studies have demonstrated that UA can reduce the production of pro-inflammatory cytokines, which are typically elevated during influenza infection. Compounds **46**–**53** (Figure 26, Figure 27, Figure 28, Figure 29 and Figure 30) are representative UA-based compounds with antiviral activity. However, UA derivatives against other viral pathogens such as HSV and HCV have not been reported. Experiments show that the introduction of ester at the C3 or C28 position of UA can enhance the pharmacological activity, and further modification of the position of C3 may be an effective strategy to obtain compounds with stronger activity.

In summary, the structural properties of derivatives of UA modifications at the C3 position or within the ring A and C-28 positions of the UA skeleton have been widely reported. Additionally, to discover the potential of UA derivatives, further development and more evaluation of UA derivatives for other pharmacological activities are necessary, similar to the efforts focused on anticancer UA derivatives. This review can be useful to researchers working in the field of medicinal chemistry, as it will aid in the design and development of novel UA-based compounds with potent therapeutic activities.

## Figures and Tables

**Table 1:** The anti-inflammatory activity of various UA derivatives (**2**–**7**), including the method of modification, the tested cancer cell lines, and the observed effects.

Compounds	Modification Method	Tested Models/Assays	Observed Effects	Ref.
**2**	Incorporated piperazine, triazolone, and oxadiazole groups at the C-3 position	Ear edema model	Decreased ear swelling, reduced COX-2 expression	[43]
**3**	attached 1,2,3-triazole groups at the C-28 position	Para-xylene-induced mice ear-swelling	Reduced inflammation, reduced COX-2 expression	[61]
**4**	Incorporated a 1,2,4-triazolo[1,5-a]pyrimidine group at C-28 position.	xylene-induced ear edema model	Decreased the production of the inflammatory factors, inhibited COX-2	[61]
**5**	Incorporated an aminoguanidine moiety	xylene-induced ear edema	Reduced inflammation	[62]
**6**	Linked UA with modified gallate moieties through 1,2,3-triazole employing CuAAC 1,3-cycloaddition reactions	RAW 264.7 macrophages	Inhibited pro-inflammatory cytokines by suppressing the LPS-induced PI3K/Akt signalling pathway, suppressed mRNA levels of iNOS (*p* < 0.05) and COX-2) (*p* < 0.01).	[63]
**7**	introduced a 1,2,3-triazoles moiety, 1,2,4-triazoles moiety or a nitroimidazoles ring to the C-28 of UA nucleus	xylene-induced ear edema	Inhibited HIF-1α, and COX-2.	[66]

**Table 2:** The anticancer activity of various UA derivatives, including the method of modification, the tested cancer cell lines, and the observed effects.

Compounds	Modification Method	Tested Cancer Cell Lines	Observed Effects	Ref.
**8**	Used Jones reagent to deliver the C-3 oxidized UA derivative then incorporated benzaldehyde and indole.	U251 (Glioblastoma)	Suppressed the growth of glioma cells, triggered apoptosis, and halted cell cycle progression by down-regulating metabolic pathways	[81]
**9**	Introduced a secondary amine at position C-3 of a cleaved ring-A	NSCLC (Lung cancer)	Induced apoptosis and autophagy	[82]
**10**	Linked the triphenylphosphonium group to a UA at the C-28 position through the hydrophobic n-butyl or hydrophilic triethylene glycol spacer	MCF-7 (Breast adenocarcinoma) and TET21N (Neuroblastoma)	Induced mitochondria-dependent apoptosis	[83]
**11**	Reacted UA with 1,2-dibro-moethane, 1,3-dibromopropane, 1,4-dibromobutane or butyl bromide in DMF in the presence of K_2_CO_3_, and then reacted with corresponding amines to yield the targeted compounds.	Bcap-37 (Breast cancer) and MGC-803 (Gastric cancer)	Induced apoptosis on MGC-803 cells,	[84]
**12**	UA was coupled with 1,4-dibromo-butane in the presence of K_2_CO_3_ and KI in DMF. The resulting intermediate was subsequently reacted with piperazine.	A549 and H460 (Lung cancer)	Inhibited cell proliferation, induced apoptosis, Increased cell cycle arrest in the G0/G1 phase	[85]
**13**–**16**	They oxidized the UA via John reagent (PCC), introduced various bromo-alkanes at the C-28 position, and then added 1-(4-nitrophenyl)hydrazine at the C-3 position.	BEL7402 (liver cancer) and SGC7901 (Gastric cancer)	Reduced tumor growth, enhanced cytotoxicity, and inhibited the NF-*k*B pathway of tumor cells.	[86]
**17**	Fused aminoguanidine moiety at the UA skeleton.	HCT116 (Colon cancer), A549: (Lung cancer), Hep3B (Liver cancer), HeLa (Cervical cancer)	reduced HIF-1α protein levels inhibited hypoxia-induced expression of VEGF at both the mRNA and protein levels and inhibited the proliferation of cancer cells in vitro.	[38]
**18**	Incorporated hydrazide, and oxadiazole moieties into UA structure.	SMMC-7721 (Liver cancer), HeLa (Cervical cancer), MDA-MB-231 (Breast cancer)	Enhanced cytotoxicity, Induced apoptosis of HeLa cells, arrested cell cycle at the G0/G1 phase, elevated intracellular reactive oxygen species level, decreased mitochondrial membrane potential, inhibited MEK1 kinase activity, and impeded Ras/Raf/ MEK/ERK transduction pathway	[87]
**21**	Incorporated hydrazide derivatives into UA structure.	SMMC-7721 (Liver cancer), HeLa (Cervical cancer), MDA-MB-231 (Breast cancer)	Induced apoptosis in MDA-MB-231 cell lines in a dose-dependent manner. Additionally, promoted G0/G1 phase arrest in MDA-MB-231 cell lines.	[88]
**24**, **25**	They oxidized UA using Jone’s reagent, followed by treatment with NH_2_-OH·HCl. The resulting intermediate was then reacted with Ac_2_O. This intermediate was subsequently condensed with suitable amino and phenol compounds in the presence of triethylamine.	HeLa (Cervical cancer), HepG2 (Liver cancer), BGC-823(Gastric cancer)	Inhibited cell proliferation, Enhanced cytotoxicity	[89]
**26**–**28**	Designed novel indolequinone derivatives of UA-bearing ester, hydrazide, or amide moieties	MCF-7 (Breast cancer), HeLa (Cervical cancer), HepG2 (Liver cancer)	Enhanced cytotoxicity, suppresses the migration of MCF-7 cells, elevates intracellular reactive oxygen species (ROS) levels, and decreases mitochondrial membrane potential. upregulated Bax, cleaved caspase-3/9, cleaved PARP levels and downregulated Bcl-2 level of MCF-7 cells, inhibited cell proliferation	[40]
**29**–**33**	UA was modified by introducing a tetrazole moiety, with the tetrazole group directly attached to the nitrogen atom of the amide group at the C-28 position. The C-3 hydroxy group was either left unmodified, oxidized, esterified, or converted to hydrazine	Hep3B cells (Liver cancer)	Inhibited the HIF-1α	[39]
**34**, **35**	Combined UA with two different azole types (1,3,4- oxadiazole and 1,2,3- triazole or 1,2,5- oxadiazole and 1,2,3- triazole) at different positions of UA.	MCF-7 (Breast cancer), HepG2 (Liver cancer), A549 (Lung cancer), **U-87MG** (Glioblastoma)	Enhanced cytotoxicity	[90]
**36**	Incorporated different constituents of the benzylidene at C-2	HepG2 (Liver cancer), HT-29 (Colon cancer), A549 (Lung cancer)	Induced apoptosis via arrest of the cycle at the G1 phase and mitochondria-mediated pathway. Enhanced cytotoxicity	[92]
**37**	Acetylation of the hydroxyl group at the C-3 position. Introduction of 2-chloroethanol at the C-28 position. Addition of methanesulfonyl chloride (MsCl) in pyridine. Reaction with piperazine. Oxidation with PCC. Introduction of 4-fluorobenzyl bromide at the piperazine moiety	MKN45 (Gastric cancer)	Decreased the apoptosis regulator (BCL2/BAX) ratio, disrupted mitochondrial potential, induced apoptosis, and suppressed the growth of Hela xenografts in nude mice.	[93]
**38**, **39**	Converted the UA into C-28-amino-functionalized derivatives	HeLa, Jurkat, Hek293, K562, and U937	Inducted the cell cycle arrest at the S-phase and apoptosis.	[94]
**40**	UA was treated with acetic anhydride in dry pyridine under the 4-dimethylamino pyridine. The 3-acetyl UA was treated with oxalyl chloride to produce an intermediary 28-acyl chloride. This compound was then mixed with piperazine to produce the targeted compound.	SUM149PT (Breast cancer), HCC1937(Breast cancer),	Suppressed cell proliferation and triggered apoptosis in both cell lines	[95]
**41**	Acylated the C-3(OH) position. Converted the carboxylic group at the C-8 position oxalyl chloride ((CO)_2_C_l2_). The intermediated was reacted with hexamethylenediamine (H_2_N(CH_2_)_6_NH_2_). Then reacted with 3,4,5-triacetoxybenzoic acid to form the amide bond.	A549 (Lung cancer), HepG2 (Liver cancer) KOV3 (Ovarian cance) T24 (Bladder cancer)	Inhibited the binding of NF-κB to DNA, suppressed NF-κB activation, inhibited A549 cell migration in vitro, and arrested A549 cell line at the G1 phase.	[36]
**42**	Methylated the C-28 carboxylic group of UA using diazomethane to produce the methyl ester	HepG2, Hep3B and HA22T/VGH (Liver cancer)	Inhibited cell growth and induced an inhibition of NF-κB activation in hepatocellular carcinoma cell lines	[33]

**Table 3:** The summary of the antibacterial activity of compounds (**43**–**45**), including the method of modification, the tested bacterial strains, and the observed effects.

Compounds	Modification Method	Bacterial Strain	Effects	Ref.
**43**	UA was reacted with acetic anhydride (Ac_2_O) in pyridine at room temperature for 24 h to yield compound **54**	*K. pneumoniae* (ATCC 10031) *Shigella flexneri* (ATCC 12022) *E. coli* (ATCC 25922)	Enhanced antibacterial activity against *Shigella flexneri* and *E. coli*, a multidrug-resistant clinical isolate from sputum	[109]
**44**	Hybridization of UA with hydrazide and 1,3,4-oxadiazole groups	*S. mutans* ATCC 25175, *Fusobacterium nucleatum* ATCC 10953	Showed significant antibacterial activity against *S. mutans*	[116]
**45**	The commercial anhydride was added to UA in pyridine (CH_2_Cl_2_, 2 mL) to form an ester derivative	*E. faecalis*, *S. epidermidis* and *S. aureus*	An antibiofilm activity against *S. aureus* without any effect on mammalian cells.	[115]

**Table 4:** The antiviral activity of various UA derivatives, including the method of modification, the tested viruses, and the observed effects.

Compounds	Modification Method	Target Virus	Notes	Ref.
**48**	Modified UA as P2 ligands and phenylsulfonamide as P2′ ligands	HIV-1	Demonstrated HIV-1 protease inhibition, exhibiting 67 times greater inhibitory activity compared to its precursor, UA	[118]
**49**, **50**	Attached the privileged fragment 2-(piperidin-1-yl)ethan- 1-amine or its bioisosteric surrogate 2-(1,3- oxazinan-3-yl)ethan-1- amine into UA by a crucial amide linker	H5N1, PR/8 (H1N1), JX/312 (H3N2)	50 Inhibited infection of H1-, H3-, and H5-typed influenza A viruses by interfering with the viral hemagglutinin	[122]
**51**	Modified the C-28 position of UA saponins via conjugation with a series of amide derivatives	H5N1	Inhibited influenza A virus replication	[123]
**52**	Coupled lamivudine and UA with ethyl chloroacetate through an amide and ester linkage		Had the dual action of anti-hepatitis B virus activity and hepatoprotective effects against acute liver injury	[124]
**53**	UA was oxidized using Jones’ reagent. The resulting compound was then reacted with hydroxylamine hydrochloride (NH_2_OH·HCl). The intermediate was further reacted with acrylonitrile (CH_2_CHCN).	Human papillomavirus type 11	Inhibited human papillomavirus type 11	[14]

**Table 5:** The antioxidant activity of UA derivatives (**54**–**56**), including the method of modification, the tested models or assays, and the observed effects.

Compounds	Modification Method	Antioxidant Assay	Notes	Ref.
**54**	UA was reacted with acetic anhydride (Ac_2_O) in pyridine at room temperature for 24 h to yield compound **54**	DPPH Radical Scavenging Assay	Strong antioxidant activity	[109]
**55**, **56**	Hybridization of UA with hydrazide and 1,3,4-oxadiazole groups	DPPH Radical and ABTS Radical Scavenging Assay	High antioxidant activity compared to ascorbic acid	[116]

**Table 6:** The antidiabetic activity of various UA derivatives, including the method of modification, the tested models or assays, and the observed effects.

Compounds	Modification Method	Assay/Model Used	Notes	Ref.
**57**–**60**	UA was esterified in anhydrous pyridine with different anhydrides	α-Glucosidase Inhibition Assay	Strong inhibition of α-glucosidase compared to acarbose, the positive control	[28]
**61**–**63**	For compounds **61** and **62** the reaction was initiated by adding a base to the UA at 0 °C in (CH_2_Cl_2_) or (THF). Then, an acyl or alkyl halide was added at C-3, and the mixture was subjected to microwave irradiation and refluxed.For compound **63**, UA was reacted with (LiAlH_4_) in tetrahydrofuran (THF) for 8 h	PTP-1B inhibition assay	Significant inhibitory activity on PTP-1B enzyme in a reversible manner	[29]
**66**, **67**	UA was esterified in anhydrous pyridine with different anhydrides	Glucose Uptake in L6 Myotubes	Displays an inhibitory effect on 2-NBDG uptake through inhibiting SGLT-1 and GLUT-2 transporter protein expression in Caco-2 cells	[128]
**68**–**71**	Conjugation of hydrophilic and polar groups at C-3 and/or C-28 position	α-Glucosidase Inhibition Assay	Inhibited α-glucosidase through a mixed-type inhibition, while compounds **70** and **71** exhibited a non-competitive inhibition mechanism	[129]

## Data Availability

Not applicable.
